# The Genomic and Transcriptomic Analyses of *Floccularia luteovirens*, a Rare Edible Fungus in the Qinghai–Tibet Plateau, Provide Insights into the Taxonomy Placement and Fruiting Body Formation

**DOI:** 10.3390/jof7110887

**Published:** 2021-10-20

**Authors:** Zhengjie Liu, Hongyun Lu, Xinglin Zhang, Qihe Chen

**Affiliations:** 1 Department of Food Science and Nutrition, Zhejiang University, Hangzhou 310058, China; liuzhengjie@zju.edu.cn (Z.L.); luhongyun@zju.edu.cn (H.L.); xinglinzhang@zju.edu.cn (X.Z.); 2 College of Food and Pharmacy, Zhejiang Ocean University, Zhoushan 316022, China

**Keywords:** biosynthesis gene clusters (BGCs), *Floccularia luteovirens*, fruiting body formation, genome, phylogeny

## Abstract

*Floccularia luteovirens* is a famous and precious edible mushroom (Huang Mogu) on the Qinghai–Tibet plateau that has a unique flavor and remarkable medical functions. Herein, we report a reference-grade 27 Mb genome of *F. luteovirens* containing 7068 protein-coding genes. The genome component and gene functions were predicted. Genome ontology enrichment and pathway analyses indicated the potential production capacity for terpenoids, polyketides and polysaccharides. Moreover, 16 putative gene clusters and 145 genes coding for secondary metabolites were obtained, including guadinomine and melleolides. In addition, phylogenetic and comparative genomic analyses shed light on the precise classification of *F. luteovirens* suggesting that it belongs to the genus *Floccularia* instead of *Armillaria*. RNA-sequencing and comparative transcriptomic analysis revealed differentially expressed genes during four developmental stages of *F. luteovirens*, that of which helps to identify important genes regulating fruiting body formation for strain modification. This study will provide insight into artificial cultivation and increase the production of useful metabolites.

## 1. Introduction

*Floccularia luteovirens* (also known as *Armillaria luteovirens* (Alb. and Schwein.) Sacc.), as a well-known unique Chinese medicinal and edible basidiomycete, is mainly distributed in the meadows and grasslands of Qinghai–Tibet plateau (Figure S1A,B) [1]. As a traditional Tibetan medicine, *F. luteovirens* is frequently used for the treatment of neurasthenia, dizziness, insomnia, headaches, infantile convulsions and numbness in limbs. It has been a tribute since recommended by Empress Dowager Cixi in the Qing Dynasty. To the best of our knowledge, as the unique local species, *F. luteovirens* is found to have significant biological activities against radiation, hypoxia, and cancer in the previous studies [2,3,4]. Therefore, the chemical compounds with officinal value in *F. luteovirens* have been analyzed for their pharmacological properties [4,5,6,7,8]. However, our understanding of *F. luteovirens* biology is limited despite its medicinal value due to little information about this species. There is scarce literature describing the genomic composition, genetic characteristics, and metabolism genes for secondary metabolites in *F. luteovirens*. Besides, as reported in previous studies, the taxonomic status of *F. luteovirens* has not been fully elucidated, especially at the genomic level. Some researchers even used the name “*Armillaria luteovirens*” in the articles of *F. luteovirens* [2,4,5,6,7,9]. Moreover, many nucleotide sequences of *F. luteovirens* were submitted to the NCBI Nucleotide database with the name “*Armillaria luteovirens*”. As a result, research reports on *F. luteovirens* were confusing. It was only in recent years that researchers have begun to use the name *F. luteovirens* in general [10,11,12]. NCBI removed the webpage of *Armillaria luteovirens* in the Taxonomy Database in 2019, which means researchers reached an agreement that this rare edible fungus belongs to the genus *Floccularia* instead of *Armillaria*. However, there were few articles about the species classification of *F. luteovirens*. Therefore, it is obligated to fully elucidate the taxonomic status of *F. luteovirens* at the genomic level. Moreover, wild *F. luteovirens* fruiting bodies are becoming scarce resources. In the main producing area Qilian County, the production of *Floccularia luteovirens* was 6000 kg in 2017 and the production declined in recent years due to the environmental damage in the growing region. In addition, the harvest of wild *F. luteovirens* fruiting bodies is annual and the artificial cultivation of *F. luteovirens* has been unsuccessful to harvest fruiting bodies. Thus, it is necessary to elucidate the molecular mechanism of fruiting body formation in this special mushroom.

In this work, we report a 27 Mb draft genome sequence of *F. luteovirens* C10. The genome information was used to analyze the genome composition and gene function. Considering the medicinal value of *F. luteovirens*, the gene clusters associated with bioactive secondary metabolites were also detected. Due to the long-standing debate focusing on the strain classification of *F. luteovirens*, we tried to demonstrate its taxonomic status at the genomic level. Lastly, we used comparative transcriptome analysis to identify the candidate genes influencing *F. luteovirens* fruiting body formation. The genome and transcriptome sequence of *F. luteovirens* would provide insight into this precious macrofungus and will be useful for developing a strategy for artificial cultivation and increasing the production of useful metabolites.

## 2. Materials and Methods

### 2.1. Isolation of F. luteovirens and Cultivation Method

Mycelia of *F. luteovirens* were isolated and screened from an *F. luteovirens* fruiting body growing in the steppe of Qilian Mountain, Qinghai, China. This culture collection which had been originally isolated in our laboratory, was named FLZJUC10 (strain C10 in this work). Mycelium was maintained on potato dextrose agar (PDA) slant at 25 °C and monthly subcultured. For transcriptome samples, we selected four major developmental stages of *F. luteovirens* which were mycelium (MY), primordium (PR), young fruiting body (YF), and mature fruiting body (MF). To RNA-seq each growing stage, three individual samples were used as three biological replicas.

### 2.2. Library Preparation and High-Throughput Sequencing

For genome sequencing, genomic DNA was extracted using a modified cetrimonium bromide (CTAB) procedure [13]. Libraries for single-molecule real-time (SMRT) sequencing were constructed with an insert size of 20 kb using the SMRTbell Template Prep kit 1.0 (Pacific Biosciences, Menlo Park, CA, USA). Subsequently, the genome of C10 was sequenced using PacBio Sequel platform by single-molecule, real-time (SMRT) technology [14] and Illumina NovaSeq PE150 at the Novogene Bioinformatics Technology Co., Ltd. (Beijing, China). The low-quality reads were filtered by the SMRT Link v5.1.0 [14,15] and the filtered reads were assembled to generate one contig without gaps.

For transcriptome sequencing, the samples were frozen in liquid nitrogen and ground to powder. Subsequently, the total RNA of the samples was prepared with Trizol reagent according to the manufacturer’s instructions. The RNA-seq transcriptome library was prepared following the TruSeq^TM^ RNA Sample Preparation Kit from Illumina (San Diego, CA, USA). Subsequently, RNA-seq was performed with the Illumina Novaseq 6000 (2 × 150 bp read length) at Majorbio technology Inc. (Shanghai, China).

### 2.3. Transcriptome Reads Mapping

The raw paired-end reads from RNA-seq were trimmed and quality controlled by SeqPrep (https://github.com/jstjohn/SeqPrep, accessed on 25 August 2018) and Sickle (https://github.com/najoshi/sickle, accessed on 25 August 2018) with default parameters. Then clean reads were separately aligned to reference genome with orientation mode using TopHat (http://tophat.cbcb.umd.edu/, Version 2.1.1, accessed on 13 October 2018) [16] software. The C10 genome sequence was used as the reference genome. The mapping criteria of bowtie was as follows: sequencing reads should be uniquely matched to the genome allowing up to two mismatches, without insertions or deletions. Then, the region of the gene was expanded following depths of sites and the operon was obtained. Besides, the whole genome was split into multiple 15 kb windows that share 5 kb. New transcribed regions were defined as more than two consecutive windows without overlapped regions of gene, where at least two reads per window mapped in the same orientation.

### 2.4. Analysis of Genome Composition

Genome component prediction included the prediction of the coding genes, repetitive sequences, and non-coding RNA. The available steps were proceeded as follows: The Augustus 2.7 program [17] was employed to retrieve the related coding genes. The interspersed repetitive sequences were predicted using the RepeatMasker [18] (http://www.repeatmasker.org/, accessed on 30 October 2018). The tandem repeats were analyzed by the TRF (tandem repeats finder) [19]. Transfer RNA (tRNA) genes were predicted by the tRNAscan-SE [20]. Ribosomal RNA (rRNA) genes were analyzed by the rRNAmmer [21]. sRNA, snRNA and miRNA were predicted by BLAST against the Rfam database [22].

### 2.5. Gene Functional Annotation

We used eight databases to predict gene functions. They were GO (Gene Ontology, http://geneontology.org/, accessed on 26 March 2019) [23], KEGG (Kyoto Encyclopedia of Genes and Genomes, https://www.kegg.jp/, accessed on 24 March 2019) [24,25], KOG (Clusters of Orthologous Groups, http://www.ncbi.nlm.nih.gov/COG/, accessed on 25 March 2019) [26], NR (Non-Redundant Protein Database, https://www.ncbi.nlm.nih.gov/protein/, accessed on 26 March 2019) [27], TCDB (Transporter Classification Database, http://www.tcdb.org, accessed on 27 March 2019), P450 Database (http://p450.riceblast.snu.ac.kr/cyp.php, accessed on 27 March 2019) [28], and Swiss-Prot (https://www.uniprot.org/, accessed on 28 March 2019) [29], respectively. A whole-genome Blast search (E-value less than 1 × 10^−5^, minimal alignment length percentage larger than 40%) was performed against above-mentioned databases. The secretory proteins were predicted by the SignalP database (http://www.cbs.dtu.dk/services/SignalP/, Version 4.1, accessed on 27 March 2019) [30]. Meanwhile, we analyzed the secondary metabolism gene clusters by the antiSMASH 5.0 (https://fungismash.secondarymetabolites.org/, accessed on 28 March 2019) [31]. Carbohydrate-active enzymes were predicted by the Carbohydrate-Active enZYmes Database (http://www.cazy.org/, accessed on 30 February 2019) [32,33]. For transcriptome, the genes functional annotation referred to the result of the genome sequence.

### 2.6. Phylogenetic and Comparative Genomic Analyses

Phylogenetic and comparative genomic analyses included the construction of the phylogenetic tree and the genomic synteny analysis. Phylogenetic analysis of *F. luteovirens* C10 based on the ITS and LSU sequences (retrieved from genome sequencing data). Phylogenetic tree derived from the ITS and LSU sequences analysis of strain C10 and related fungi which were selected from the NR annotation result. The evolutionary history was inferred using the maximum likelihood method [34]. The percentage of replicate trees in which the associated taxa clustered together in the bootstrap test (1000 replicates) are shown next to the branches [35]. *Calocera viscosa* was used as the outgroup. The tree was drawn to scale, with branch lengths in the same units as those of the evolutionary distances used to infer the phylogenetic tree. The evolutionary distances were computed using the Kimura 2-parameter method [36]. Evolutionary analyses were conducted with MEGA 7 [37]. Genomic synteny of C10 and reference genomes was analyzed using GATA [38].

### 2.7. Differential Expression Analysis and Functional Enrichment

To identify DEGs (differential expression genes) between two different samples, the expression level of each transcript was calculated according to the fragments per kilobase of exon per million mapped reads (FPKM) method. RSEM [39] was used to quantify gene abundances. R statistical package software EdgeR (Empirical Analysis of Digital Gene Expression in R) [40] was utilized for differential expression analysis. In addition, functional-enrichment analysis including GO and KEGG were performed to identify which DEGs were significantly enriched in GO terms and metabolic pathways at Bonferroni-corrected *p*-value ≤ 0.05 compared with the whole-transcriptome background. GO functional enrichment and KEGG pathway analysis were carried out by Goatools (https://github.com/tanghaibao/Goatools, accessed on 30 May 2019) and KOBAS 2.1.1 (http://kobas.cbi.pku.edu.cn/download.php, accessed on 30 May 2019) [41].

### 2.8. RT-qPCR Validation

Total RNA obtained in the transcriptome sequencing experiment was used to synthesize cDNA with Prime Script^TM^ RT Reagent Kit with gDNA Eraser (TaKaRa, Shiga, Japan). Primers for RT-qPCR were designed in Primer-BLAST [42] of NCBI (Table S1). Beta-tubulin and GAPDH (glyceraldehyde-3-phosphate dehydrogenase) were the internal reference genes. The RT-qPCR was performed using QuantStudio 3 system (Life Technologies, Camarillo, CA, USA) and the expression levels of genes were calculated by the 2^−∆∆CT^ method.

### 2.9. Data Availability

The genome data in this study have been submitted to GenBank’s Sequence Read Archive (SRA) database under accession number ASM973921v1. The RNA-seq data have been deposited in NCBI under accession number PRJNA616182. The alignment files for the phylogenetic tree had been deposited in TreeBASE under accession number 26577.

## 3. Results

### 3.1. Strain Isolation and General Genome Features of F. luteovirens

*Floccularia luteovirens* strain C10 was isolated from a typical *F. luteovirens* fruiting body (Figure S1C). The hyphae of colonies on PDA plates were white, fluff-like, and with aerial growth (Figure S1D). Using a light microscope, the hyphae of C10 were hyaline, septate, and branching, and the clamp connections of hyphae were observed clearly and no spores were observed (Figure S1E). The ITS sequence obtained with primers ITS4 and ITS5 was used to determine the identity in a BLAST nucleotide search of NCBI and exactly matched the ITS region of *Armillaria luteovirens* (GenBank accession GCA_009739215.1) with 100% similarity. The search result indicated that the strain C10 was a pure culture strain of *F. luteovirens* (*A. luteovirens*).

**Figure 1:**
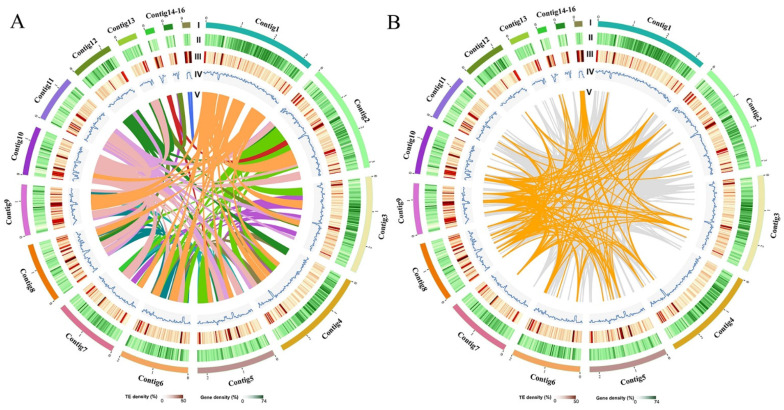
Distribution of *F. luteovirens* genomic features. (Circles I in **A**,**B**) Circular representation of the pseudomolecule (Length > 0.08 Mb). (Circles II–IV in **A**,**B**) gene density (30 kb window), percentage of repeats (30 kb window), and GC content (30 kb window). (Circle V in **A**) Each linking line in the center of the circle connects a pair of homologous genes. (Circle V in **B**) Genome duplication: regions sharing more than 90% sequence similarity over 1 kb are connected by grey lines; those with more than 90% similarity over 5 kb are connected by orange lines.

With the PacBio Sequel and Illumina NovaSeq PE150 sequencer, raw data containing 378,128 reads and 5213.4 million bases were obtained. The low-quality reads were filtered by the SMRT Link 5.1.0 and the filtered reads were assembled to generate 24 polished contigs (Figures S2 and S3). According to the coverage and the GC content, the assembly was estimated and optimized, and finally, the draft genome sequence of *F. luteovirens* C10 was obtained. The genome sequence was 27 Mb and consisted of 23 contigs with an N50 of 2.3 Mb and a GC content of 43.54% (Table 1 and Figures S4 and S5). Previous studies [43] reported that the assembled genome size of *F. luteovirens* was 28.8 Mb, and comprising 183 contigs with a N50 contig size of 571 kb and a GC content of 43.36%. It indicated that this study provided a more accurate genome assembly of *F. luteovirens*. After contigs optimized, 16 contigs-containing distribution maps of *F. luteovirens* genomic features were constructed (Figure 1).

As to genome composition, 7068 coding genes (Figure S6), 93 tRNA genes, 7 rRNA genes, and 11 snRNA genes were predicted, and the relative abundances of the different tRNA genes are shown in Figure S7. The interspersed repetitive sequences represent approximately 2.27% of the genome, including 1701 long terminal repeats, 395 DNA transposons, 444 long interspersed repeated segments, 10 short interspersed repeated segments. Comparing to interspersed repetitive sequences, there are 2362 tandem repeat sequences (repeat size 1–509 bp), including 2027 minisatellite DNAs (repeat size 10–60 bp) and 132 microsatellite DNAs (repeat size 2–6 bp) (Tables S2–S4). The interspersed repetitive sequences content of *F. luteovirens* was lower than medicinal mushroom *Lignosus rhinocerotis* which was reported as 4.01% of the assembled genome. The genome size and number of predicted genes are consistent with five other medicinal mushrooms (Table S5). All the assembly data indicated that whole genome allows a detailed analysis of the gene content, phylogeny, and metabolic pathway of *F. luteovirens*.

### 3.2. Gene Function Annotation and Analysis

**Figure 2:**
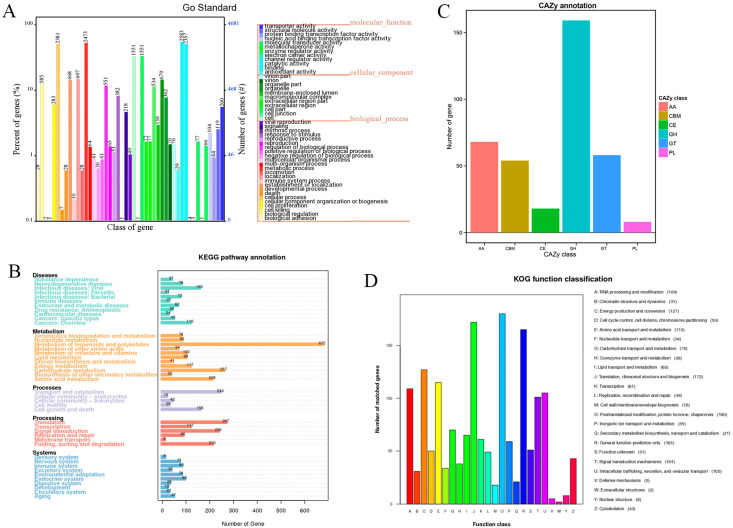
Gene function annotation of *F. luteovirens* C10 genome. (**A**) GO enrichment analysis of annotated genes exist in C10. (**B**) KEGG pathway annotation of the genome of C10. (**C**) Annotation of C10 by CAZy databases. (**D**) KOG function classification of annotated genes in C10. Distribution of predicted proteins from *F. luteovirens* genome according to functional class by Eukaryotic Clusters of Orthologs (KOG) database.

The result of genome functional annotation was summarized in Table S6. In the analysis of GO classification, 4680 genes were annotated with the three main categories: biological process, cellular component, and molecular function, respectively (Figure 2A). The identified coding proteins associated with biological process are more than molecular function and cellular component proteins. In the KEGG pathway annotation, the 5854 predicated genes could be divided into 43 categories based on their functions (Figure 2B). Among these categories, 672 genes associated with the metabolism of terpenoids and polyketides accounted for the largest proportion, which indicates that *F. luteovirens* was a potential resource for terpenoids and polyketides biosynthesis. Besides, 257 genes associated with carbohydrate metabolism could be the reason for *F. luteovirens* being rich in polysaccharides [2,3,8]. Chen et al. [44] reported the genomic analyses of *Hericium erinaceus*, which was also a famous medicinal and edible basidiomycete. In this genome, only 60 genes were found in terpenoids and polyketides biosynthesis while carbohydrate metabolism contained the highest gene number (479 genes). In KOG analysis, the 1532 predicated genes can be divided into 24 categories, according to their functions (Figure 2D). Among these categories, the number of genes related to posttranslational modification, protein turnover, chaperones (O), translation, ribosomal structure, and biogenesis (J), energy production and conversion (C), and amino acid transport and metabolism (E) were more than the other function-related genes. In the CAZy annotation, 365 candidate carbohydrate-active enzyme genes (CAZymes) were identified in the genome of the C10 strain. Interestingly, the glycoside hydrolase (159) dominated in the classes of CAZymes (Figure 2C), followed by 54 carbohydrate-binding modules, 18 carbohydrate esterases, 58 glycosyl transferases, 8 polysaccharide lyases, and 68 auxiliary activities enzymes. The CAZymes profile in *F. luteovirens* was also compared to those of seven other fungi, of which one: ectomycorrhizal fungus (*Laccaria bicolor*), four: white-rot fungi (*Lentinula edodes*, *Flammulina velutipes*, *Ganoderma lucidum*, and *Phanerochaete chrysosporium*), and two: brown-rot fungi (*Antrodia cinnamomea* and *Postia placenta*) (Table S7).

### 3.3. Cytochrome P450s and Transporters

**Figure 3:**
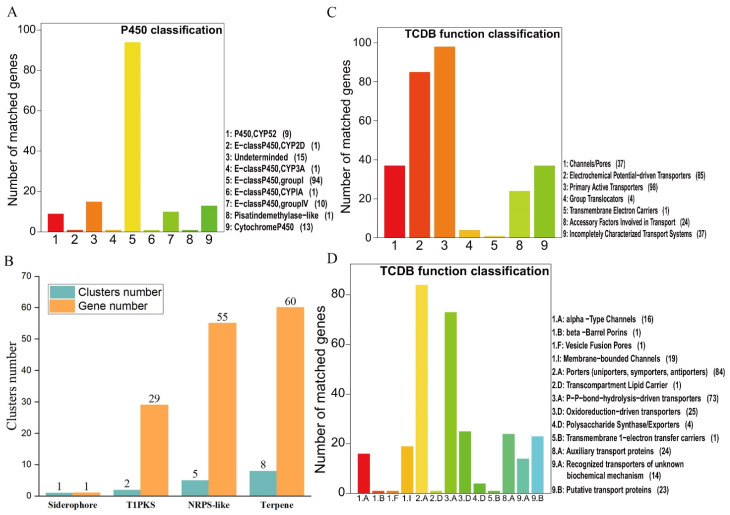
The classification of cytochrome p450, BGCs and transporters of the *F. luteovirens* C10 genome. (**A**) Cytochrome p450 classification. (**B**) The composition of BGCs of C10. (**C**,**D**) Transporter function classification.

According to previous studies, cytochrome P450s and transporters had important roles in the biosynthesis and transportation of fungal metabolites [45,46,47]. Thus, we identified the genes belonging to these families in the *F. luteovirens* genome. We identified a total of 145 CYP450 sequences, which could be classified into nine classes according to standardized CYP nomenclature (Figure 3A and Table S8) [48]. Among these classes, the group I class was found to have the greatest number of genes (94 genes). As to transporters, a total of 286 transport proteins belonging to 103 families were identified in *F. luteovirens* (Figure 3C,D and Table S9). The data of these transporters classification showed that 16 transporters belonged to the major facilitator superfamily (MFS) and 9 transporters belonged to the ATP-binding cassette (ABC) superfamily. It was found that the MFS transporters participated in secondary metabolism, and the ATP-binding cassette transporters were involved in the transport of polysaccharides and lipids [49].

### 3.4. The Composition of Biosynthesis Gene Clusters (BGCs)

**Figure 4:**
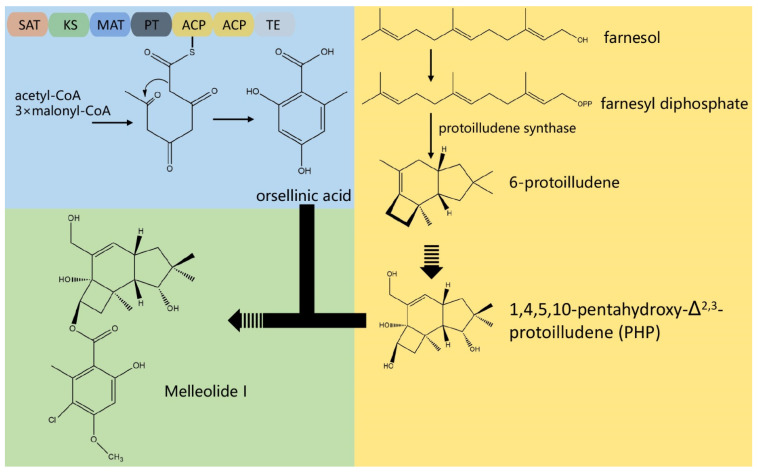
Representative secondary metabolites and biosynthesis pathways of melleolides of *Floccularia luteovirens*.

In the process of screening BGCs with antiSMASH 5.0, 16 putative gene clusters and 145 genes coding for secondary metabolites were obtained (Figure 3B). There were one siderophore BGC, two T1pks (type I PKS cluster) BGCs, five NRPS (Nonribosomal peptide synthetase cluster)-like BGCs, and eight terpene BGCs. Nevertheless, most of BGCs were unable to determine their roles with only a few similar genes. There was a guadinomine BGC with 14% similarity and a melleolides BGC with 100% similarity (Table S10) which belonged to the type I PKS cluster. However, considering that guadinomine was generally thought to be produced by bacteria [50], the guadinomine BGC with 14% similarity still worthy of further investigation. On the other hand, the melleolides BGC with 100% similarity was noteworthy. Melleolides, a family of sesquiterpene aryl esters, are natural products with various bioactivities including anti-inflammation, antimicrobial, antifungal, and cytotoxicity against cancer cells [51,52,53]. According to previous studies [54,55,56], the biosynthetic pathway of melleolides was demonstrated as shown in Figure 4. Among 14 genes in the melleolides BGC (Figure S8), the genes A5734, A5736 (armB), and A5737 (armH4) encode cytochrome P450, orsellinic acid synthase, and flavin-dependent halogenase, respectively, which had a direct relation with the melleolides biosynthesis [55,56]. With previous methods [52,57,58], melleolide I was isolated and identified from the liquid fungal cultures of C10 (Figure S9) according to the available standards, which confirmed the biosynthesis of melleolides.

### 3.5. Phylogenetic Analysis of Strain C10

**Figure 5:**
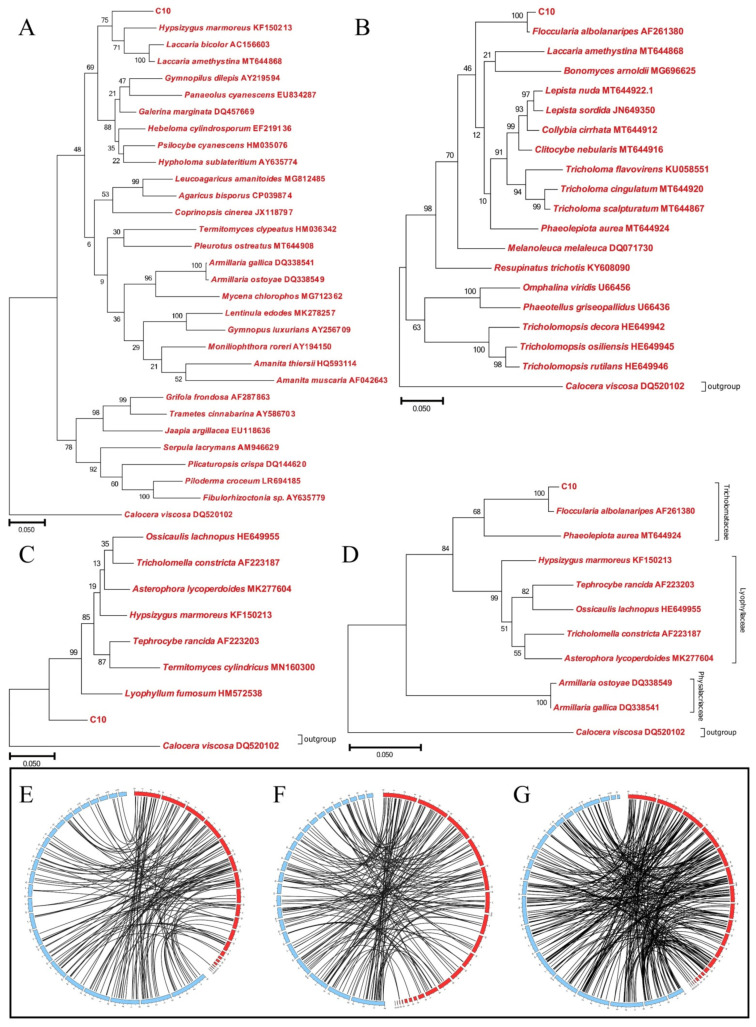
Phylogenetic analysis of *F. luteovirens* C10 based on the ITS and LSU sequences and whole genome synteny analysis of *F. luteovirens* C10 with *Armillaria gallica*, *Hypsizygus marmoreus* and *Lepista sordida*. (**A**) Phylogenetic tree derived from the ITS sequence analysis of strain C10 and related fungi from the result of NR annotation; (**B**) phylogenetic tree derived from the ITS sequence analysis of strain C10 and selected fungi belonging to family *Tricholomataceae*; (**C**) phylogenetic tree of strain C10 and selected fungi belonging to family *Lyophyllaceae*; (**D**) phylogenetic tree of C10 strain and selected fungi belonging to families *Tricholomataceae* and *Lyophyllaceae*. *Calocera viscosa* was used as the outgroup. (**E**) *Armillaria gallica* vs. C10; (**F**) *Hypsizygus marmoreus* vs. C10; (**G**) *Lepista sordida* vs. C10. Red represent *F. luteovirens* C10. Black lines link syntenic genes.

In the NR database, 6136 genes were annotated by BlastP with E-values of  ≤1 × 10^−5^. The top 30 species with high similarities were listed in Figure S10 according to the number of the matched protein-coding genes. Interestingly, the species most similar to C10 does not belong to the genus *Armillaria*. In the NR annotation, there were three matched species belonging to the genus *Armillaria, namely A. ostoyae*, *A. solidipes*, and *A. gallica*, in which the number of the matched genes was only 56, 55, and 43, respectively. In contrast, the top 3 species closer to C10 were *Hypsizygus marmoreus* (1263 matched genes), *Laccaria amethystina* (863 matched genes), and *Galerina marginata* (638 matched genes). As well known, in the past decades *F. luteovirens* (also known as *Armillaria luteo-virens Sacc.*) was considered to belong to the genus *Armillaria*. However, more researchers have recommended reclassifying this species in recent years according to the phylogenetic analysis [1]. Therefore, we used the ITS sequence to determine the phylogeny of the C10 strain. Phylogenetic tree was constructed to demonstrate its taxonomic status. In Figure 5A, C10 was clustered together with *Hypsizygus marmoreus* and *Termitomyces* sp. with 92% similarity. Besides, C10 was evolutionally close to *Laccaria amethystina* and *Laccaria bicolor* which belong to the family *Tricholomataceae*. Interestingly, the phylogenetic tree suggested that C10 was evolutionarily distant from *Armillaria ostoyae* and *Armillaria gallica* which belong to family *Physalacriaceae* genus *Armillaria*. Therefore, we selected several species belonging to the family *Tricholomataceae* and *Lyophyllaceae* to construct phylogenetic trees, separately. In Figure 5B, C10 was clustered together with *Floccularia albolanaripes* with 100% similarity. On the contrary, C10 was evolutionarily distant from the species of family *Lyophyllaceae* (Figure 5C). To compare the phylogeny of C10 between *Tricholomataceae* and *Lyophyllaceae*, we constructed a phylogenetic tree with several species belonging to the family *Tricholomataceae* and *Lyophyllaceae*. The analysis results suggested that C10 was evolutionally close to the fungi of *Tricholomataceae* and was clustered together with *Floccularia albolanaripes* with 100% similarity (Figure 5D). Though the phylogenetic trees did not match with the result of NR exactly, both of the results of phylogenetic trees and NR implied that C10 belongs to the genus *Floccularia* instead of *Armillaria* (Table S11). The alignment files for phylogenetic tree had been deposited in TreeBASE under accession number 26577.

Synteny analysis of strain C10 genome with other three fungal genomes, *Armillaria gallica* (*Physalacriaceae*), *Hypsizygus marmoreus* (*Lyophyllaceae*), and *Lepista sordida* (*Tricholomataceae*), revealed that the C10 genome displayed different synteny with those fungi (Figure 5E–G). Of all the sequenced genomes, C10 showed higher synteny with *Lepista sordida* than others, which was similar to the result of phylogenetic trees. At least, the findings of synteny analysis indicated that C10 was evolutionarily distant from the species of *Armillaria*.

### 3.6. Transcriptome Sequencing and Assembly

**Figure 6:**
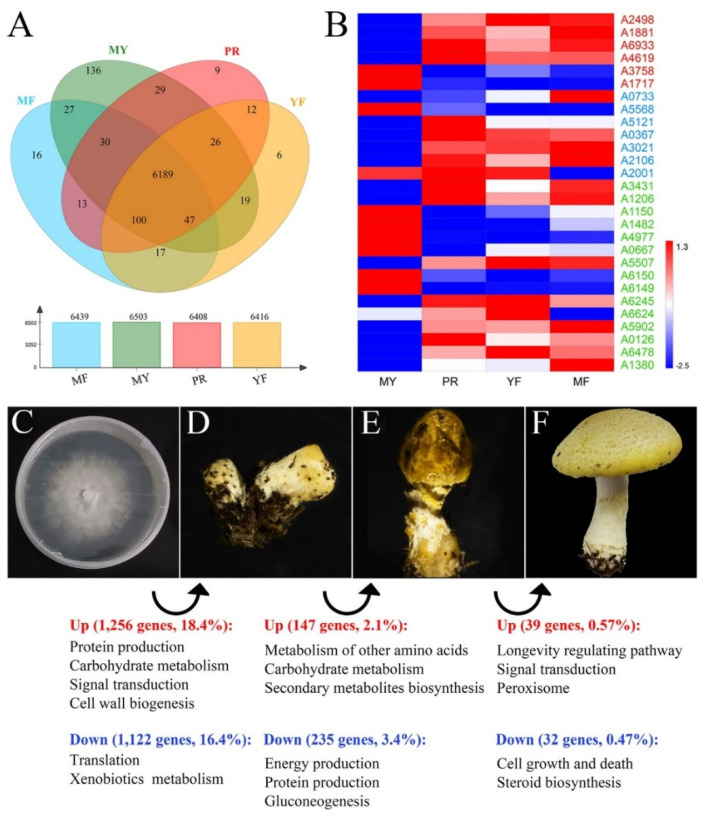
The differentially expressed genes (DEGs) during *F. luteovirens* development. (**A**) Venn diagrams depicting the genes expressed across the different developmental stages. (**B**) Heatmap of differential gene expression associated with fruiting formation in *F. luteovirens*. (**C**–**F**) The typical developmental stages in the life cycle of *F. luteovirens* were illustrated. (**C**) The vegetative mycelia formed a fluffy white layer on top of the PDA plates. (**D**) Aerial hyphae interacted with each other to form primordia. (**E**,**F**) These primordia further differentiated into fruiting bodies. Enrichment analysis showed that particular functional terms were over-represented in genes that were up- or down-regulated during a developmental transition.

To characterize the expressed sequences of *F. luteovirens*, we conducted transcriptome sequencing of four major developmental stages in *F. luteovirens* which were mycelium (MY), primordium (PR), young fruiting body (YF), and mature fruiting body (MF). Overall, total RNA was extracted from samples (Figure S11) to obtain cDNA libraries. 661,379,108 raw reads were generated from 13 cDNA libraries (Table S12). After data filtering and trimming, 655,992,276 high-quality clean reads were obtained. Subsequently, with the C10 genome sequence as the reference genome, we assembled 6860 genes. The correlation between the two samples was analyzed based on the TPM result (Figure S12). As showed in the Venn plot of gene expression analysis (Figure 6A and Figure S13), 6189 genes were co-expressed during all four developmental stages of *F. luteovirens*. In contrast, 136, 9, 6, and 16 genes were specifically expressed only during the MY, PR, YF, and MF stages, respectively.

### 3.7. The Differentially Expressed Genes (DEGs) Regulated Fruiting Body Formation

To further identify and evaluate the differentially expressed genes (DEGs), we constructed three DEG libraries to compare MY to PR, PR to YF, YF to MF. Overall, we detected 1265, 147, and 39 up-regulated DEGs and 1122, 235, and 32 down-regulated DEGs between MY and PR libraries, PR and YF libraries, YF and MF libraries, respectively (Figure 6C–F). As illustrated in Figure 6C,D, the largest number of DEGs occurred during the vegetative-to-reproductive transition stage-from MY to PR. This finding reveals that the period of MY to PR was the most active and key for fruiting body formation of *F. luteovirens*.

At first, functional enrichment analysis was conducted using all DEGs against the GO database in order to investigate DEGs involved in development and reproduction. Six genes associated with GO terms related to developmental process, reproduction, and reproductive process were identified. Among them, four genes (A2498, A1881, A6933, and A4619) showed up-regulation while two genes (A3758 and A1717) showed down-regulation during the vegetative-to-reproductive transition (Figure 6B). Notably, the gene A3758 directly associated with GO terms related to negative regulation of reproductive process showed down-regulation.

Subsequently, with the pipeline of the transcription factor database and the functionally annotated DEG libraries, we identified DEGs belonging to transcription factors (TF), which were found to be essential for growth and reproduction [59,60,61,62,63]. Two MADS-box genes (A0733 and A5568) (Figure 6B) which were transcription factors of morphogenesis were identified. The gene A0733 showed up-regulated expression while the gene A5568 down-regulated expression separately during the mycelia, primordia, and fruiting body stages. Moreover, other transcription factors were also identified, such as the genes bZIP (A5121), bHLH (A0367), prz1 (A3021), steA (A2106), and YABBY (A2001). These genes were up-regulated in either the primordia or fruiting body stage, or in both stages. Among them, the gene A2001 related to multicellular organism development was also up-regulated expression during reproductive growth.

In order to search for the functional pathways of interest involved in the fruiting process, we performed the Kyoto Encyclopedia of Genes and Genomes (KEGG) pathway analysis. The results indicated that the genes involved in the cell cycle (ko04111), ribosome (ko03010), and mitogen-activated protein kinases (MAPK) signaling (ko04011) pathways were differentially expressed significantly during reproductive growth. In the cell cycle and meiosis pathways (Figure S14), these DEGs included genes encoding sister chromatid cohesion protein 2 (SCC2, A1243), cell division cycle protein 20 (CDC20, A1114), cell division control protein 6 (CDC6, A2948), and other cell-cycle-regulating genes. In the ribosome pathway (Figure S15), genes encoded 49 ribosomal proteins showed down-regulated expressions during the period of MY to PR, which was contrary to previous reports [61,64]. This result revealed that the ribosome pathway might have a great impact on cell differentiation and development in *F. luteovirens*. In the MAPK signaling pathways (Figure S16), the up-regulated expression genes were identified which related to pheromone-dependent and starvation-dependent MAPK signaling pathways, including genes encoding Ste20, Ste7, Ptp2, Cla4, Hog1, Sko1, Hsl7, Ypd1, and Pak1.

DEGs involved in the primary carbohydrate metabolism pathway were also identified, such as the glycolysis pathway (ko00010) and the tricarboxylic acid (TCA) cycle (ko00020), in the reproductive growth of *F. luteovirens*. Most of the genes involved in the glycolysis pathway showed increased expression (Figure S17). In this pathway, the genes encoding glucose-6-phosphate 1-epimerase (A1707), aldehyde dehydrogenase (A4115), glucose-6-phosphate isomerase (A0195), pyruvate kinase (A1513), fructose-bisphosphate aldolase (A4589), and hexokinase (A5086) showed up-regulation in the fruiting process. However, the gene encoding alcohol dehydrogenase (A0550) showed down-regulation.

Ultimately, we identified the key genes in three DEG libraries which were reported to play a vital role in fruiting body formation in the previous studies [63,65,66,67,68,69,70,71,72,73]. As presented in Figure 6B, the gene encoding serine-threonine-rich membrane-anchored protein (A1206) was found to be significantly up-expressed in primordia and fruiting body. This result was consistent with literature reports [74]. Moreover, *exg1* (A3431) [65], *priB* (A5902) [63], arp9 (A5507) [67], *OAT* (A6245, A6624) [69], *ras* (A1380) [71], and the genes involved in the mTOR signaling pathway (A0126, A6478) [70] showed up-expression in the fruiting process. Meanwhile, *snf5* (A4977, A0667) [67], *NOX* (A1150, A1482) [66] and *pro1* (A6150, A6149) [68] were found to be significantly down-expressed during the vegetative-to-reproductive transition stage.

**Figure 7:**
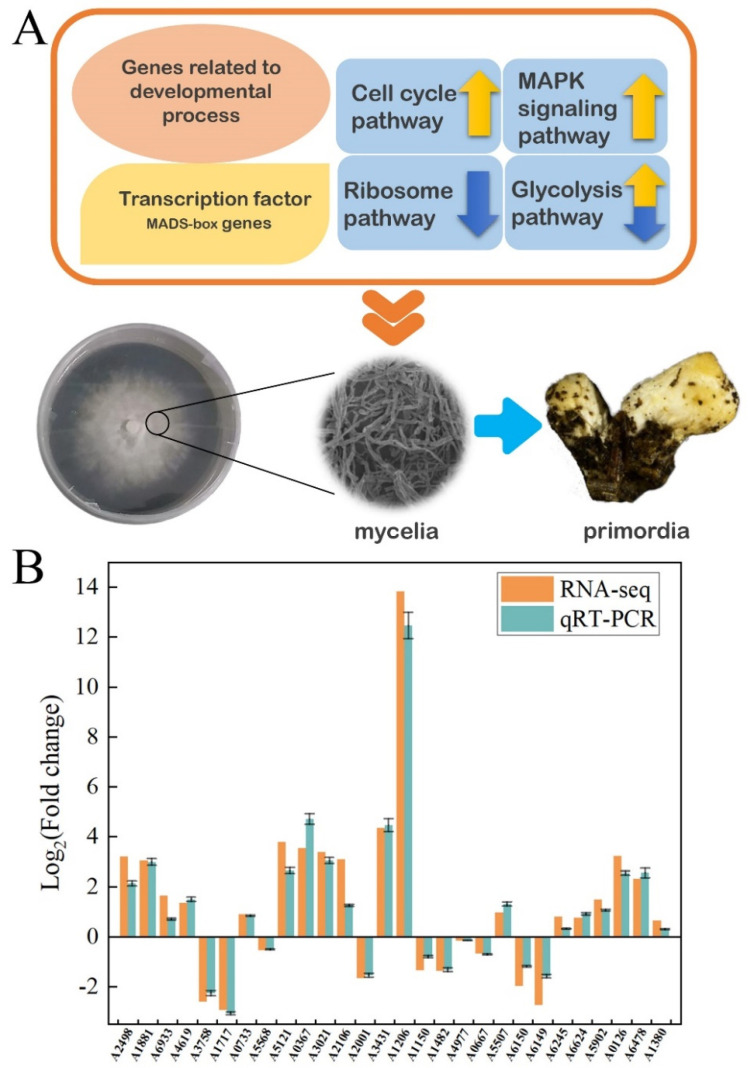
The candidate genes and pathways related to fruiting body formation. (**A**) The differentially expressed genes (DEGs) and related pathways during fruiting body formation. (**B**) Comparison of qRT-PCR and RNA-seq results of 28 genes of interest during the period of MY to PR.

At the end, quantitative real-time PCR (RT-qPCR) was used to validate the results of RNA-seq. The expression levels of 28 genes of interest (Table S13) were analyzed by RT-qPCR and the results were basically consistent with the findings of RNA-seq (Figure 7B). In general, the findings of this study indicated that the fruiting process of *F. luteovirens* was regulated by a number of genes involved in various metabolic processes, especially for serine-threonine-rich membrane-anchored protein (Figure 7A).

## 4. Discussion

Our research team has been focusing on *F. luteovirens* research for 11 years [3]. The unique growth environment and growth characteristics of *F. luteovirens* have brought high research thresholds to researchers. The mycelia of *F. luteovirens* grow slowly even in the optimized cultivation temperature of 23 °C [8]. Considering the medicinal and functional value of *F. luteovirens*, we had developed the submerged cultivation conditions for improved exopolysaccharides (EPS) production by *F. luteovirens* [8]. We also optimized the variables of the biotransformation process in order to enhance betulinic acid production from betulin catalyzed by the cultured cells of *F. luteovirens* [75]. However, the genomic background and developmental biology of this fungus were rarely reported. Herein, we report a 27 Mb draft genome sequence of *F. luteovirens* C10. The genome information would be a useful tool to investigate this rare edible fungus. Compared to the genomes of other edible fungi, *F. luteovirens* had a small genomic size [13,76,77,78,79,80]. The genomic features of *F. luteovirens* had its own unique characteristics. There were 672 genes associated with the cell metabolism of terpenoids and polyketides accounting for the largest proportion, which indicates that *F. luteovirens* was a potential resource species for terpenoids and polyketides. Likewise, 257 genes associated with carbohydrate metabolism which also accounted for a large proportion could be the reason for *F. luteovirens* being rich in polysaccharides [8]. Meanwhile, *F. luteovirens* was an ectomycorrhizal fungus, resulting in less genes associated with lignocellulose degradation than white-rot fungus and brown-rot fungus [12], such as *Lentinula edodes* [81], *Ganoderma lucidum* [45], and *Antrodia cinnamomea* [46].

In addition to the genes involved in the biosynthesis of triterpenoids and polysaccharides, *F. luteovirens* had 16 putative gene clusters involved in the biosynthesis of NRPs, PKs, and terpenes, including a melleolides BGC with 100% similarity. Melleolides were reported as natural products with more than 70 described members [82]. Previously, melleolides were isolated from *Armillaria mellea* in the beginning [83,84,85]. With the development of research, more derivatives were found [54,82,86,87,88]. The polyketide derivatives of melleolides were composed of an orsellinic acid (OA) moiety esterified to various sesquiterpene alcohols mediates mediated by non-reducing polyketide synthase [56]. According to previous studies, the first step in melleolides biosynthesis is performed by the delta_6_-protoilludene synthase PRO1 which catalyzes the cyclization of farnesyl diphosphate to protoilludane. The orsellinic acid synthase armB produces OA by condensing acetyl-CoA with 3 malonyl-CoA units in a three-round chain elongation reaction followed by a C2-C7 ring closure [56]. ArmB further catalyzes the trans-esterification of OA to the various sesquiterpene alcohols resulting from the hydroxylation of protoilludene [55,56]. The melleolides cluster in genus *Armillaria* fungi includes 5 cytochrome P450 monooxygenases, 4 NAD^+^-dependent oxidoreductases, one flavin-dependent oxidoreductase, and one O-methyltransferase (By similarity). The cytochrome P450 monooxygenases may be involved in protoilludene hydroxylation to elaborate melleolides with multiple alcohol groups, such as melleolide D, which carries alcohol functionalities at C-4, C-5, C-10, and C-13 (By similarity). The role of NAD^+^-dependent enzymes remains unknown (By similarity). Numerous melleolides, including arnamial, show 5′-*O*-methylation of the aromatic moiety which may be catalyzed by the methyltransferase encoded in the cluster (By similarity). The flavin-dependent oxidoreductase might represent the dehydrogenase yielding the aldehyde in position 1 of arnamial and other melleolides (By similarity). Finally, several halogenases localized outside of the cluster (armH1 to armH5), are able to catalyze the transfer of a single chlorine atom to the melleolide backbone, resulting in a 6′-chloromelleolide product [55]. However, unlike reported in previous studies, armH4 gene was found inside the melleolides BGC of *F. luteovirens* in addition to the genes of cytochrome P450 enzyme and armB. Meanwhile, the delta_6_-protoilludene synthase PRO1 gene was found to localize outside of the cluster and present as a multicopy gene in the genome of *F. luteovirens* which differed from the genus *Armillaria* fungi. In this study, melleolides compounds derived from *F. luteovirens* were also identified. Melleolides were natural products with various bioactivities including anti-inflammation, antibacterial, antifungal, phytotoxic activity, and cytotoxicity against cancer cells [51,52,53,54,57,58,89]. The isolation of melleolides compounds from *F. luteovirens* suggested that *F. luteovirens* may be used as a potential biosynthesis resource.

Although melleolides BGC were found both in the genome of *F. luteovirens* and *Armillaria mellea*, they had no obvious phylogenetic relationships that were noteworthy [9,12,90]. Taking the growing environment, for instance, *Armillaria mellea* is able to be distributed around the world [90] while *F. luteovirens* is a regional fungus. *Armillaria mellea* was a root rot fungus and a symbiotic strain with *Gastrodia elata* [91,92], while *F. luteovirens* was an ectomycorrhizal and fairy ring-forming fungus [12]. Unfortunately, the genome sequence of *Armillaria mellea* has not yet been published, and we are unable to investigate why *F. luteovirens* had the same melleolides BGC as *Armillaria mellea* did. As to phylogenetic analysis of *F. luteovirens* C10, with the results of NR annotation, phylogenetic trees, and synteny analysis, we considered that *F. luteovirens* was supposed to belong to the genus of *Floccularia* instead of *Armillaria* as it used to. It is noteworthy that the *Armillaria luteovirens* species in the Taxonomy Browser database (https://www.ncbi.nlm.nih.gov/taxonomy, accessed on 17 August 2019) was replaced by *F. luteovirens* (NCBI: txid493452) which belonged to *Tricholomataceae*. This approach was consistent with the results of the present study.

Moreover, we used comparative transcriptome analysis to identify candidate genes related to *F. luteovirens* fruiting body formation with high-throughput RNA-seq. In this work, six genes associated with GO terms related to the developmental process, reproduction, and reproductive process were identified and differentially expressed during the fruiting body formation process. Moreover, some transcription factors were found to be differentially expressed during the fruiting body formation period, especially the MADS-box genes. In addition, KEGG pathways were reported that are associated with fruiting body formation in previous studies [61,64,93]. Thus, we performed KEGG pathway analysis and found that the genes involved in the cell cycle (ko04111), ribosome (ko03010), MAPK signaling (ko04011), and primary carbohydrate metabolism (ko00010) pathways differentially expressed significantly during reproductive growth. Furthermore, previous studies had reported that some genes played a critical role in fruiting body formation [62,66,67,69,70,94,95,96]. Miyazaki et al. [63] found that the priB gene was up-regulated during fruiting body development in *Lentinula edodes*. Nakazawa et al. [67] reported that *Cc.snf5* was shown to cause defects in fruiting initiation in the agaricomycete *Coprinopsis cinerea*. In addition, Hsu et al. [73] underlined that cytochrome p450 genes were strongly expressed during fruiting body formation in *Antrodia cinnamomea*. Accordingly, we identified the key genes in three DEG libraries and found that the experimental findings were consistent with the above reports.

In summary, the elucidation of the *F. luteovirens* genome and transcriptome sequences would provide insights into this precious macrofungus. The genome information can be further used for comparative genomic studies with other mushroom species to unravel the evolution and growth characteristics of *F. luteovirens* which would help us to develop a strategy for artificial cultivation of *F. luteovirens*. Further, the biosynthesis of the pharmacologically active compounds produced by this medicinal fungus could be strategically exploited. Therefore, the comprehensive understanding of the *F. luteovirens* genome will pave the way for its future roles in the food industry, pharmacological, and mushroom industrial applications.

## Figures and Tables

**Table 1:** Overview of genomic features of *F. luteovirens* C10.

Attribute	Value
**Assembly summary**	
No. of contigs	23
Length of the largest contig (bp)	3,288,420
Length of the smallest contig (bp)	15,938
N50 length (bp)	2,275,160
N90 length (bp)	1,237,025
Percentage of assembly (%)	
Contigs ≥ 500 bp	100%
Contigs ≥ 1 kb	100%
**BUSCO analysis (%)**	
Complete (%)	89.3
Complete duplicated (%)	0.3
Fragmented (%)	3.8
Missing (%)	6.9
**Genomic component analysis**	
Genome size (bp)	27,003,024
GC content of genome	43.54%
Gene number	7068
Gene length	11,273,474
Average gene length (bp)	1595
% of genome (genes)	41.75%
GC content of protein-coding genes (%)	34.93%
Average protein length (aa)	531
Length of largest protein-coding gene, bp	4023
Length of smallest protein-coding gene, bp	66
Average no. of exons per gene	7
Average exon size (bp)	219
Average no. of introns per gene	6
Average intron size (bp)	68
Average size of intergenic regions (bp)	2225
Gene internal length	15,729,880
Number of tRNAs	93
Number of rRNAs	7
Secondary metabolite biosynthesis gene clusters (BGCs)	16

## Supplementary Materials

The following are available online at https://www.mdpi.com/article/10.3390/jof7110887/s1, Figure S1: The morphology of the fruiting body and hyphae of *F. luteovirens*, Figure S2: Histogram of read length distribution after filtering, Figure S3: Histogram of read score distribution after filtering, Figure S4: Association analysis statistics of GC content and sequencing depth of *F. luteovirens* C10 genome data, Figure S5: BUSCO analysis, Figure S6: Gene length distribution statistics, Figure S7: 93 tRNA predictions using tRNA Scan-SE, Figure S8: The melleolide-biosynthetic in *F. luteovirens*, Figure S9: HPLC analysis of C10 submerged-culture organic extract, Figure S10: Top30 matched species with NR annotation, Figure S11: Agarose gel electrophoresis of total RNA extracted from samples, Figure S12: Gene expression map, Figure S13: The correlation between each two samples based on TPM result, Figure S14: The vegetative-to-reproductive transition stage related pathway—Cell cycle, Figure S15: The vegetative-to-reproductive transition stage related pathway—Ribosome, Figure S16: The vegetative-to-reproductive transition stage related pathway—Mitogen-activated protein kinases (MAPK) signaling pathway, Figure S17: The reproductive growth stage related pathway—Glycolysis, Table S1: Primer sets used for quantitative real-time PCR, Table S2: Statistical result of interspersed nuclear elements, Table S3: Statistical result of tandem repeats, Table S4: Statistical result of non-coding RNA, Table S5: Genomic features of *F. luteovirens* and other 5 selected medicinal fungi, Table S6: Functional Annotation results, Table S7: Classification of putative carbohydrate metabolism proteins of *Floccularia luteovirens* and other edible fungi or model fungi based on CaZy database, Table S8: Statistical result of P450 genes, Table S9: Transporters in *F. luteovirens*, Table S10: Putative gene clusters coding for secondary metabolites in *F. luteovirens* C10, Table S11: Taxonomic status of *F. luteovirens*, Table S12: Statistics of transcriptome sequencing data, Table S13: Genes validated by RT-qPCR.
