# Supplementary material for: The Genomic and Transcriptomic Analyses of Floccularia luteovirens, a Rare Edible Fungus in the Qinghai–Tibet Plateau, Provide Insights into the Taxonomy Placement and Fruiting Body Formation
Source: J Fungi (Basel). 2021 Oct 20;7(11):887. doi: 10.3390/jof7110887 (PMC8618933; doi:10.3390/jof7110887)
Supplement: Supplementary file 1 [file jof-07-00887-s001.zip › jof-1403817-supplementary.pdf]

The genomic and transcriptomic analyses of *Floccularia luteovirens*, a rare edible fungus in the Qinghai-Tibet plateau, provides insights into the taxonomy placement and fruiting body formation

Zhengjie Liu [1,2], Hongyun Lu [1], Xinglin Zhang [1], and Qihe Chen [1,*]

[1] Department of Food Science and Nutrition, Zhejiang University, Hangzhou 310058, P. R. China; liuzhengjie@zju.edu.cn

[2] College of Food and Pharmacy, Zhejiang Ocean University, Zhoushan 316000, China;

*Correspondence author: chenqh@zju.edu.cn; Tel.: +86-0571-8698-4316

[*] ***Corresponding author:***
Qihe Chen

Yuhangtang Rd. 866

Department of Food Science and Nutrition

College of Biosystems Engineering and Food Science

Zhejiang University

Hangzhou 310058

P.R.China

Tel: +86-571-86984316

E-mail: chenqh@zju.edu.cn

Supplementary Material Guide

File Name: Supplementary Information

Description: Supplementary Figures, Supplementary Tables, Supplementary References.

Supplementary Figures

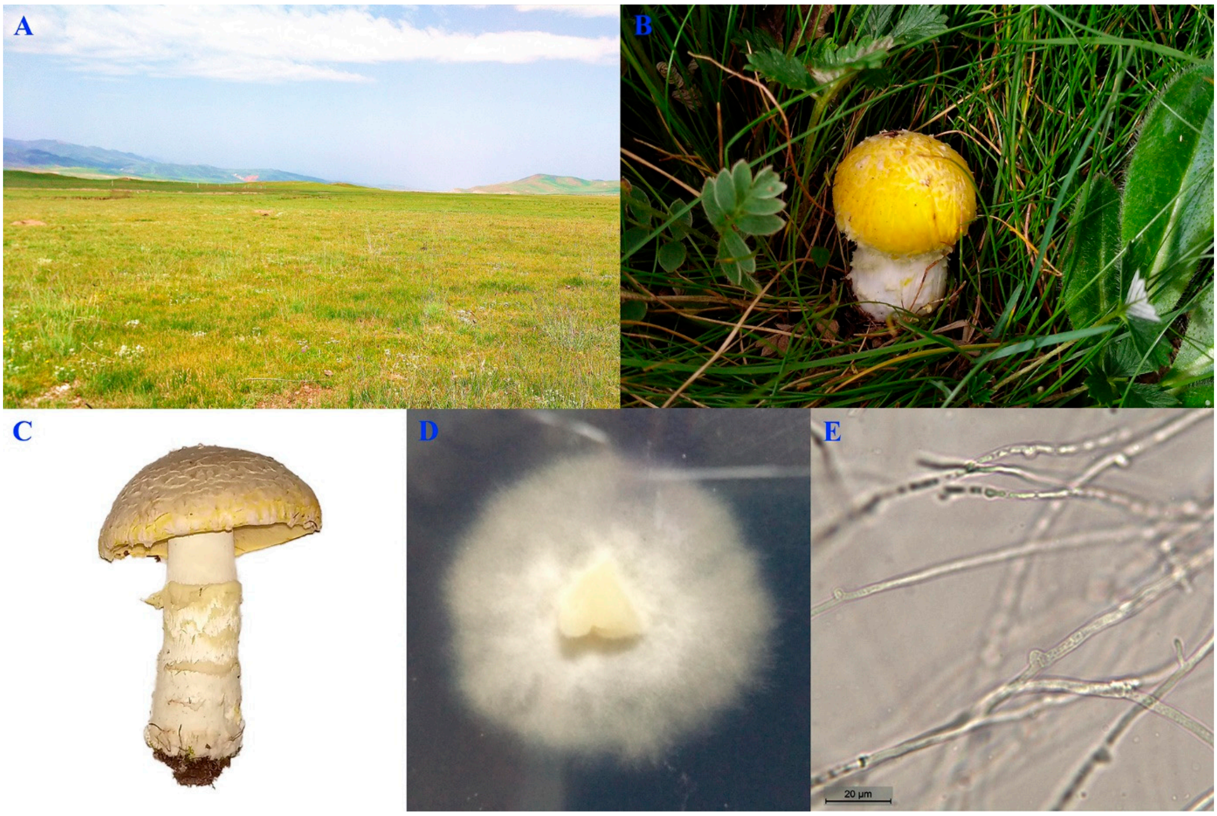

Figure S1. The morphology of the fruiting body and hyphae of *F. luteovirens*. (A-B) The inhabitable environment of *F. luteovirens* in the Qinghai–Tibet plateau. (C) The fruiting body used to isolate the strain C10; (D) Colony of *F. luteovirens* C10 isolate on PDA; (E) Morphology of isolate *F. luteovirens* C10.

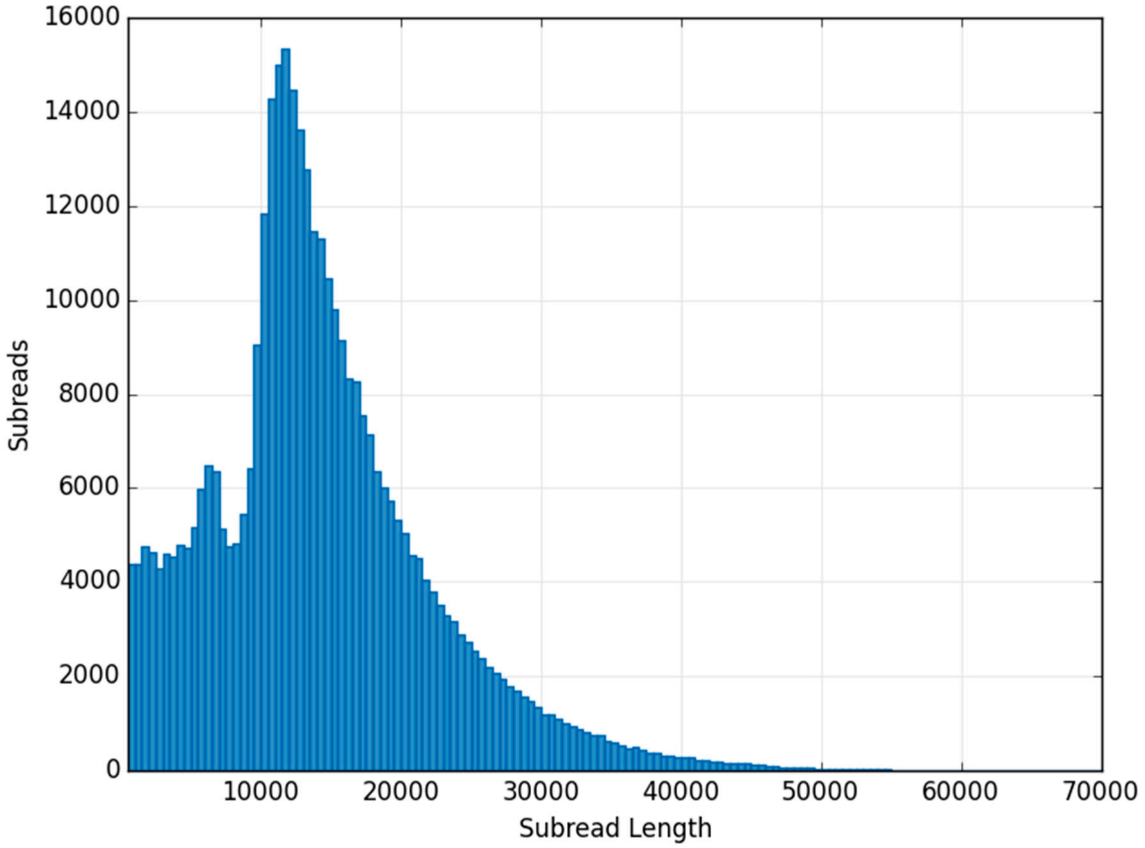

Figure S2. Histogram of read length distribution after filtering.

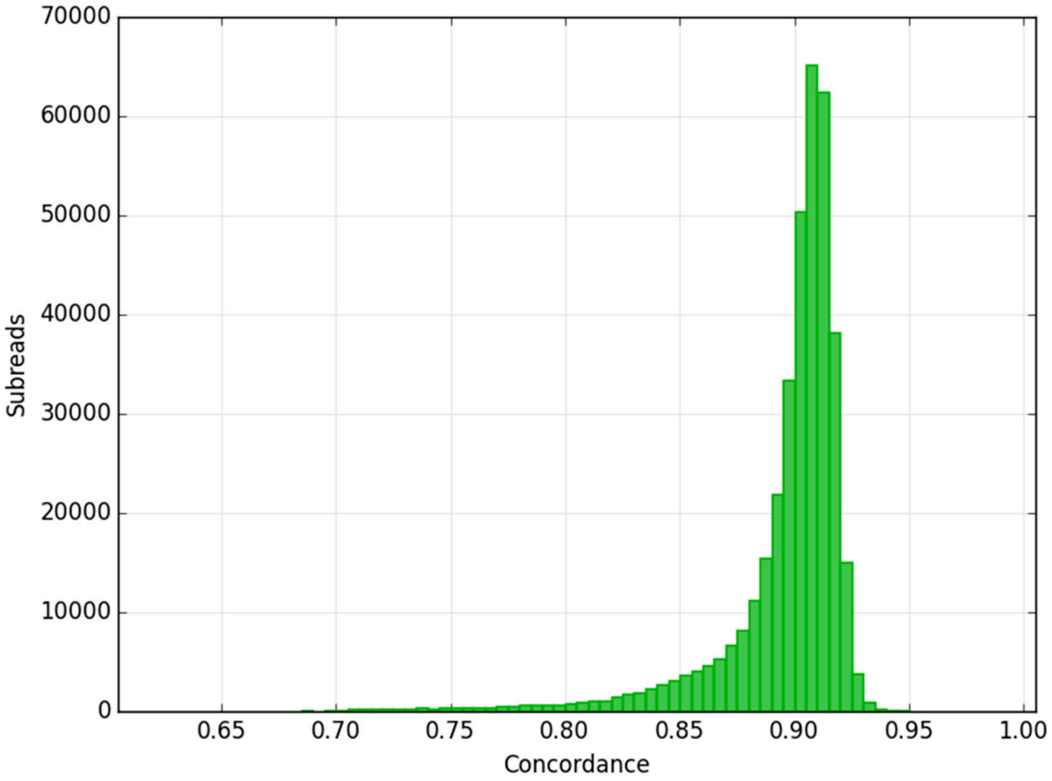

Figure S3. Histogram of read score distribution after filtering.

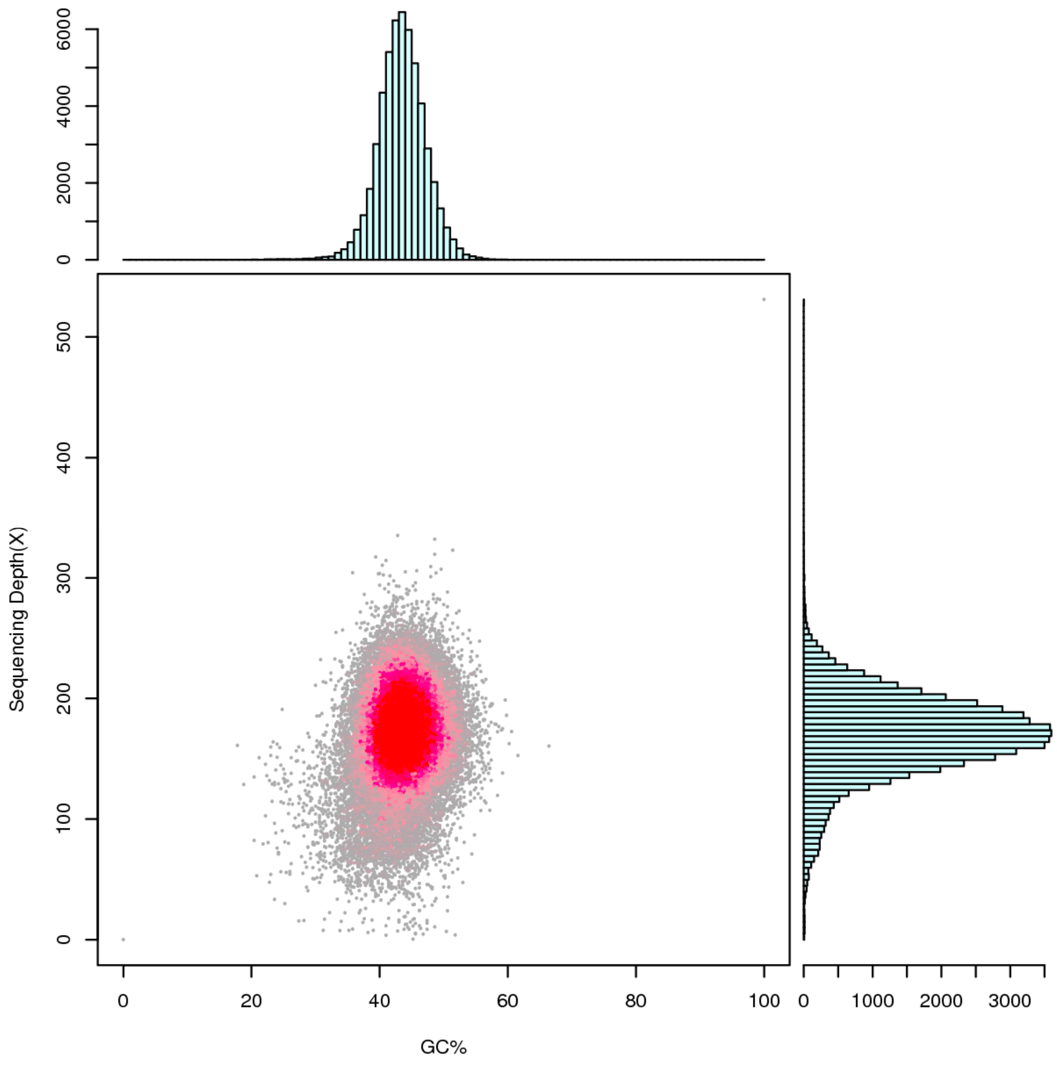

Figure S4. Association analysis statistics of GC content and sequencing depth of *F. luteovirens* C10 genome data.

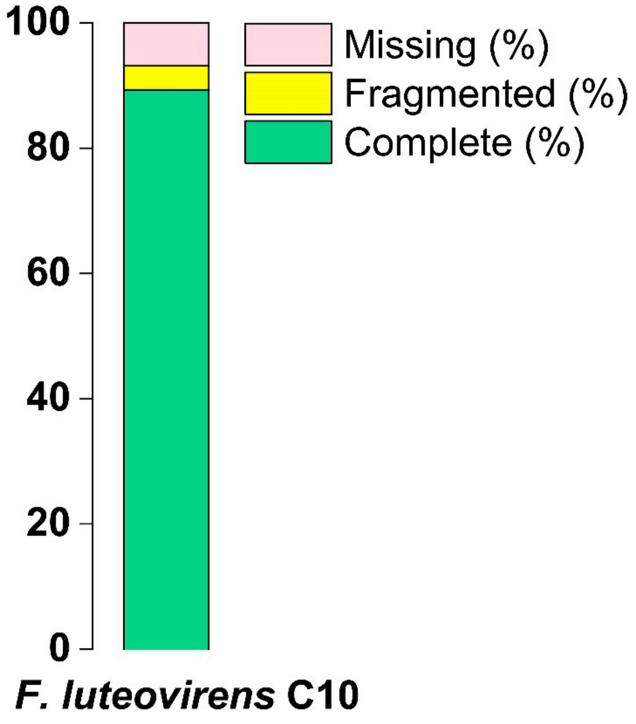

Figure S5. BUSCO analysis. Stacked bar plots showing proportions of gene sets in quality categories for *F. luteovirens* C10. The categories of genes are: i) complete single copy BUSCO: genes which match a single gene in the BUSCO reference group; ii) fragmented BUSCOs: genes only partially recovered for which the gene length exceeds the alignment length cut-off; iii) missing BUSCO: not recovered genes.

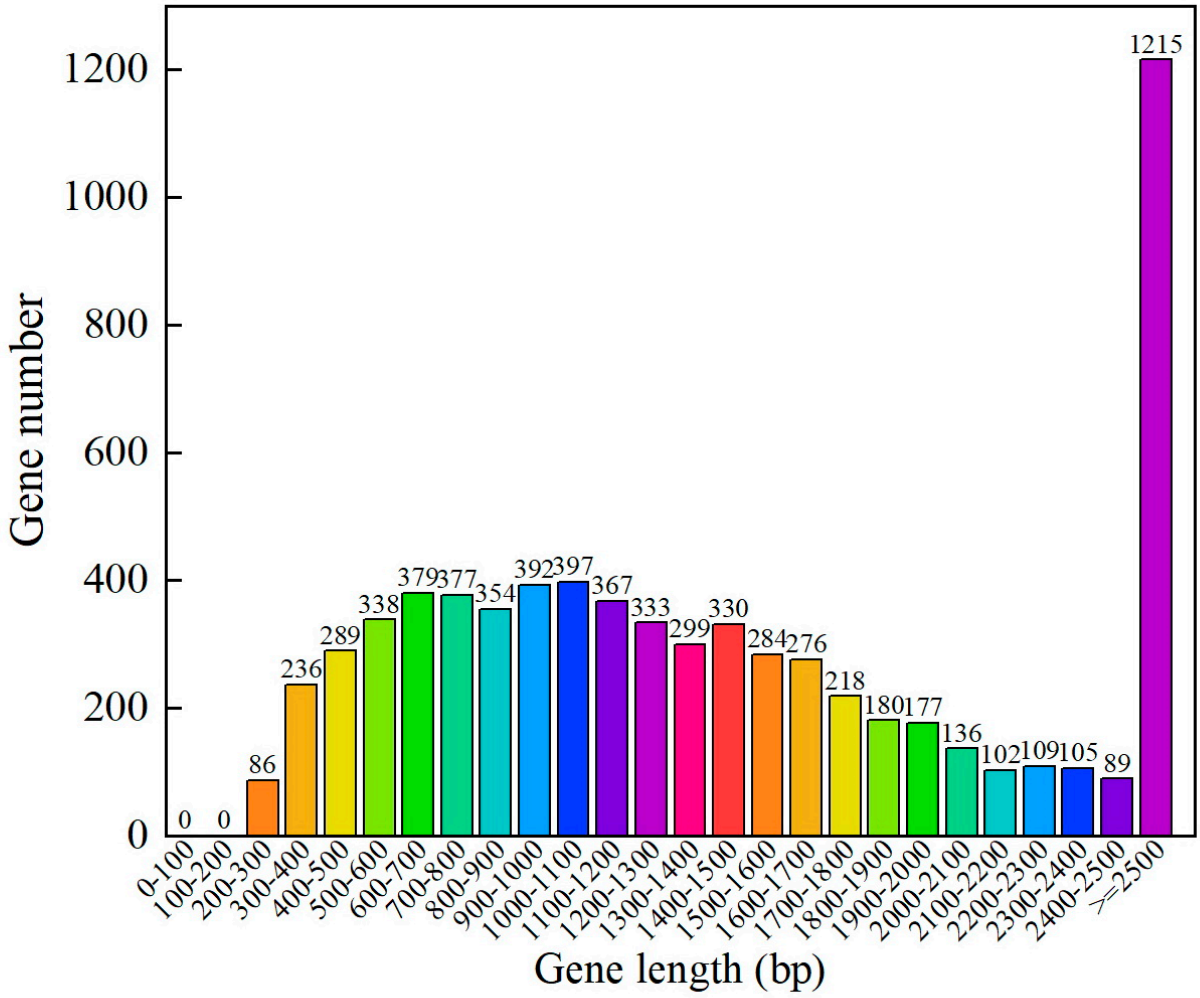

Figure S6. Gene length distribution statistics.

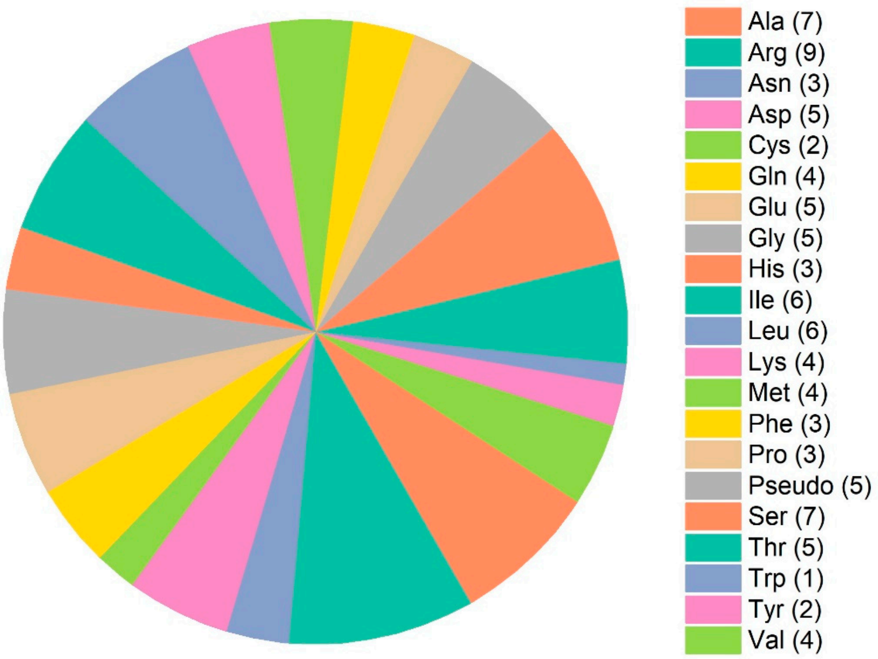

Figure S7. 93 tRNA predictions using tRNA Scan-SE.

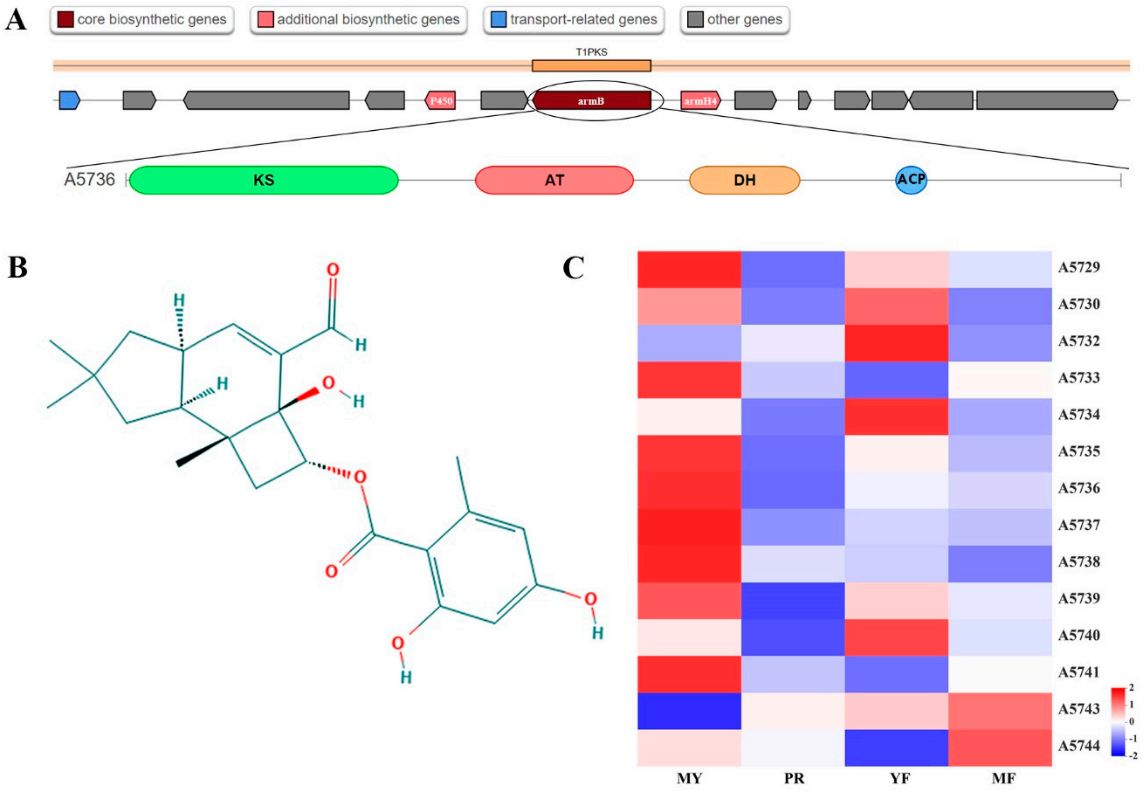

Figure S8. The melleolide-biosynthetic in *F. luteovirens*. (A) Physical map of the melleolide-biosynthetic gene cluster. The names of proteins are shown above the respective genes. Introns are not shown. (B) Chemical structure of melleolide. (C) Heatmap of differential gene expression in the melleolide-biosynthetic gene cluster during all four developmental stages of *F. luteovirens*.

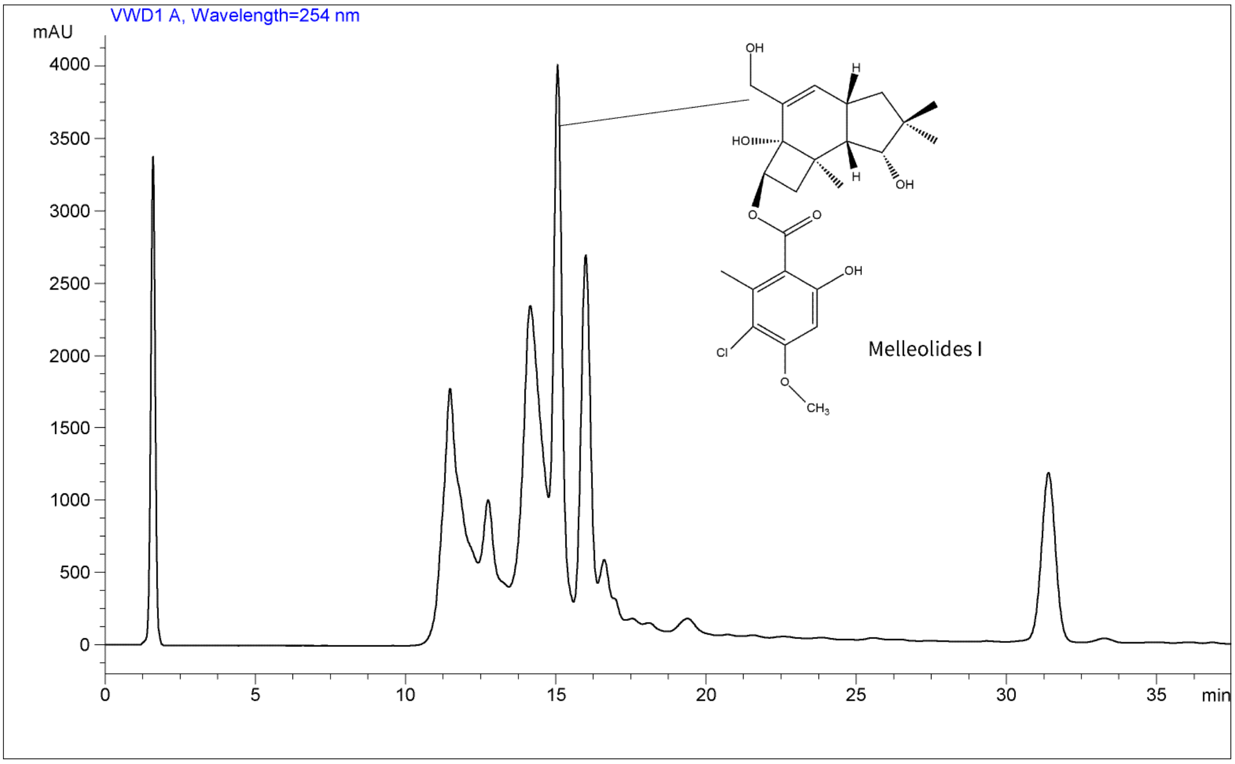

Figure S9. HPLC analysis of C10 submerged-culture organic extract. Using available standards, the observed compound peak was identified as melleolide I. mAu, milli absorption units.

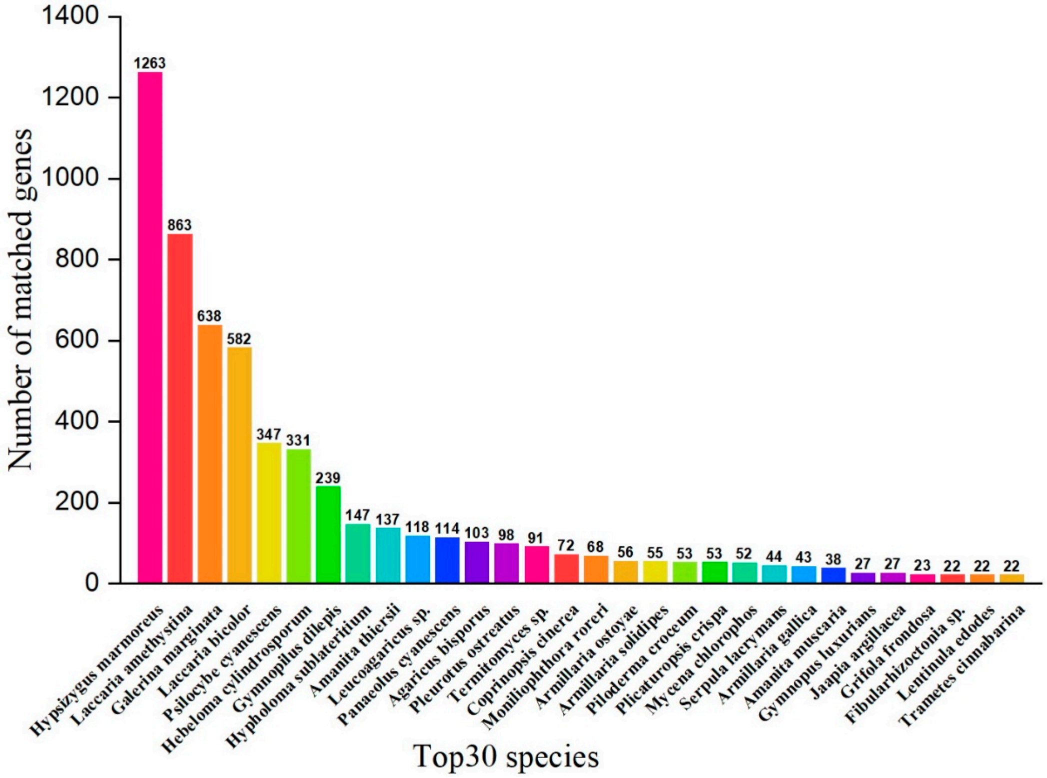

Figure S10. Top30 matched species with NR annotation.

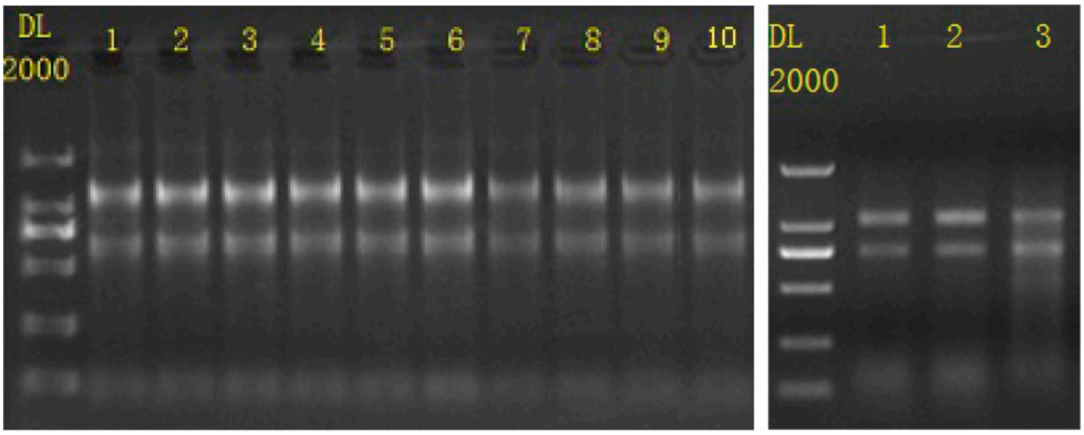

Figure S11. Agarose gel electrophoresis of total RNA extracted from samples.

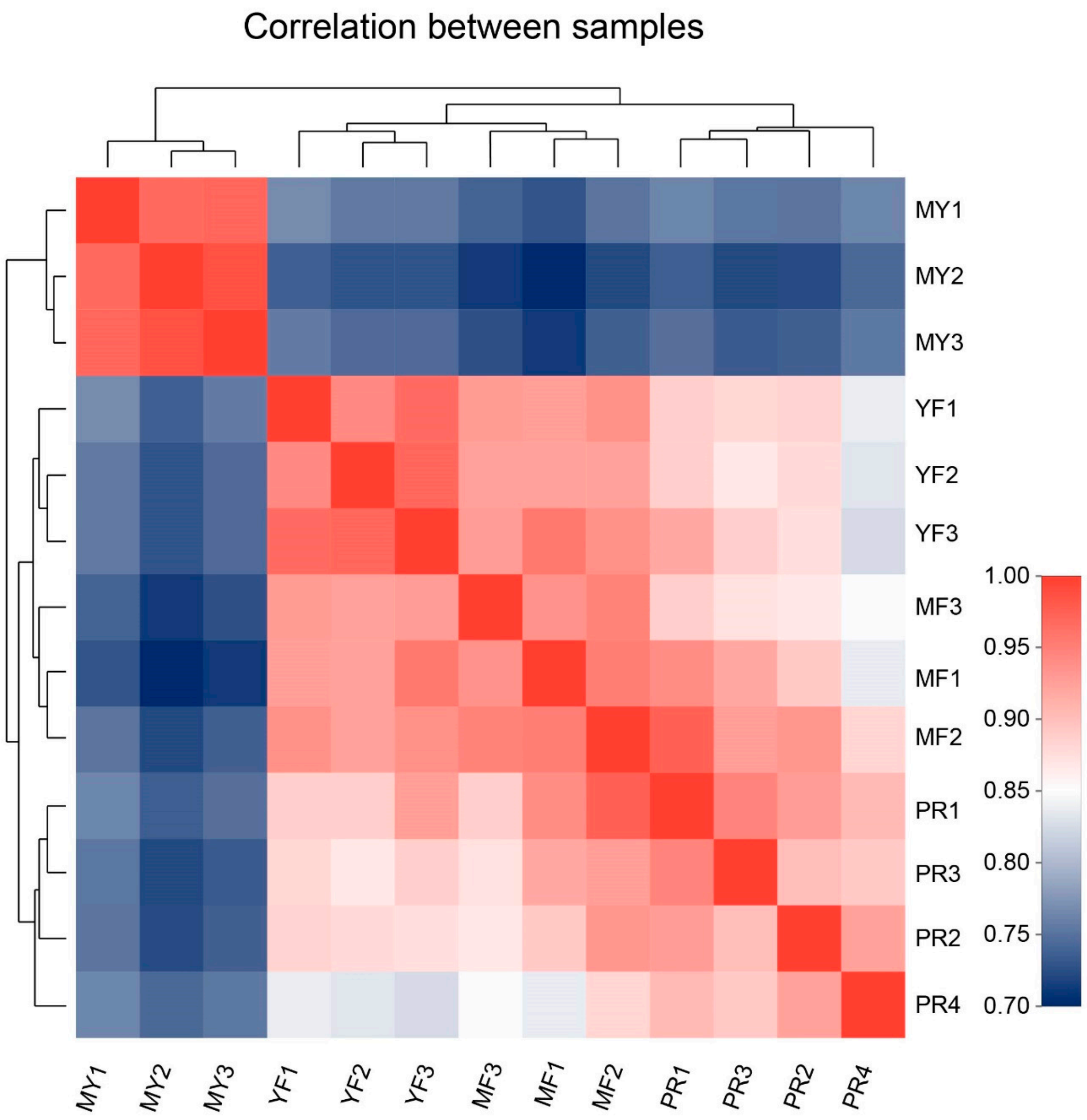

Figure S12. The correlation between each two samples based on TPM result.

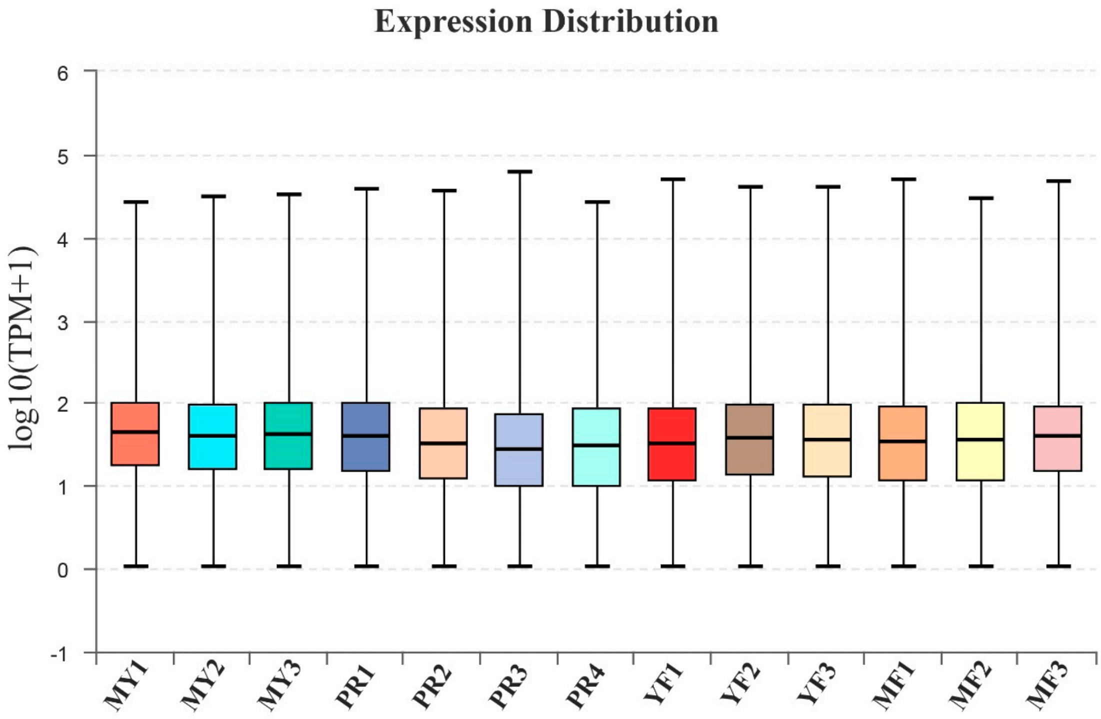

Figure S13. Gene expression map.

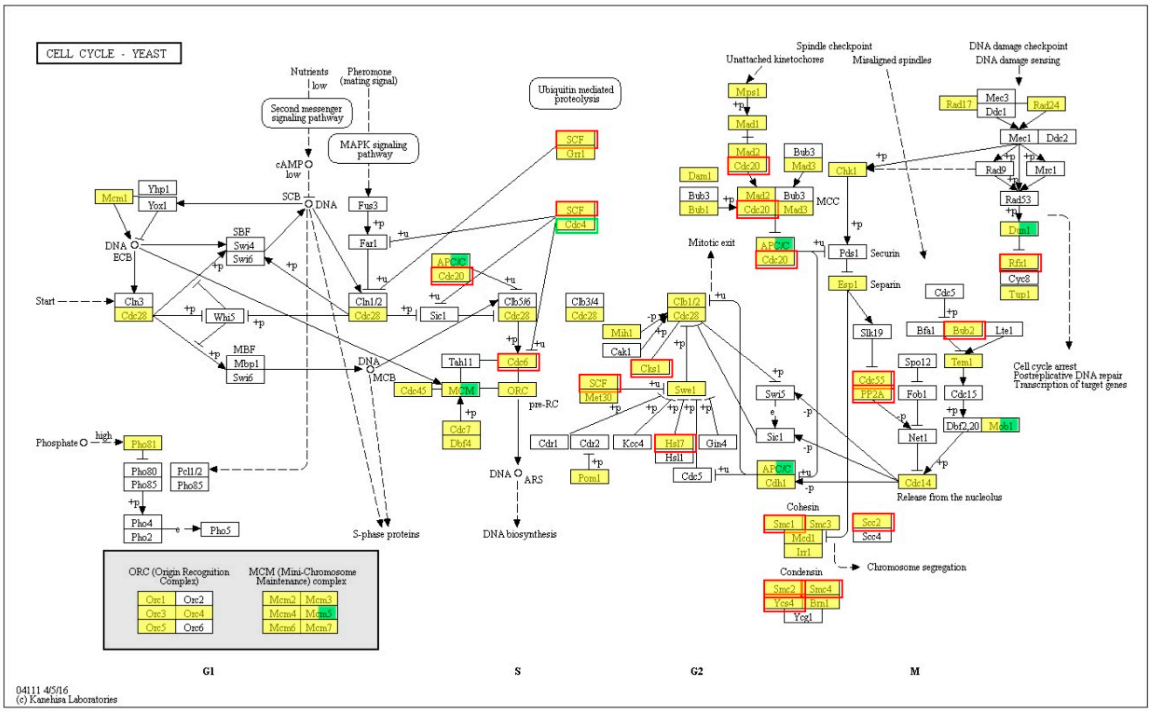

Figure S14. The vegetative-to-reproductive transition stage related pathway-Cell cycle (derived from KEGG map04111) [1-3] was identified by KEGG annotation. The red and green boxes indicate that the up-regulation and down-regulation genes.

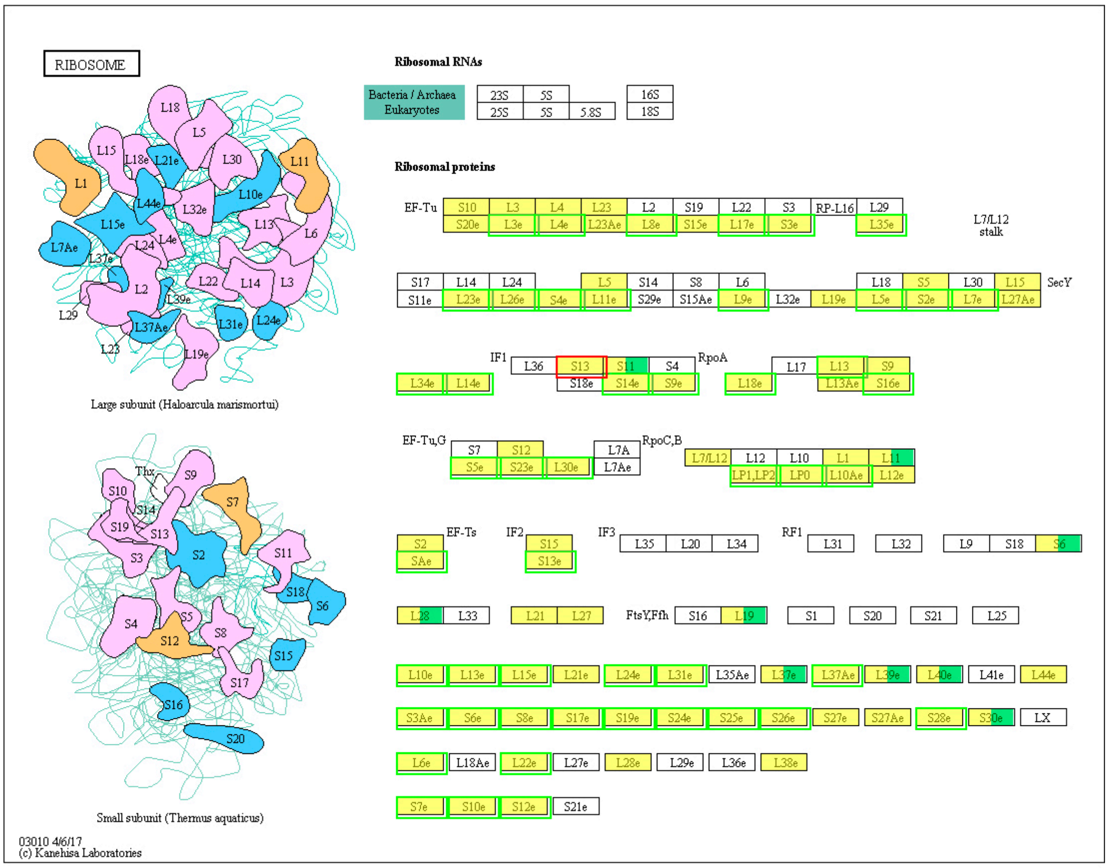

Figure S15. The vegetative-to-reproductive transition stage related pathway-Ribosome (derived from KEGG map03010) [1-3] was identified by KEGG annotation. The red and green boxes indicate that the up-regulation and down-regulation genes.

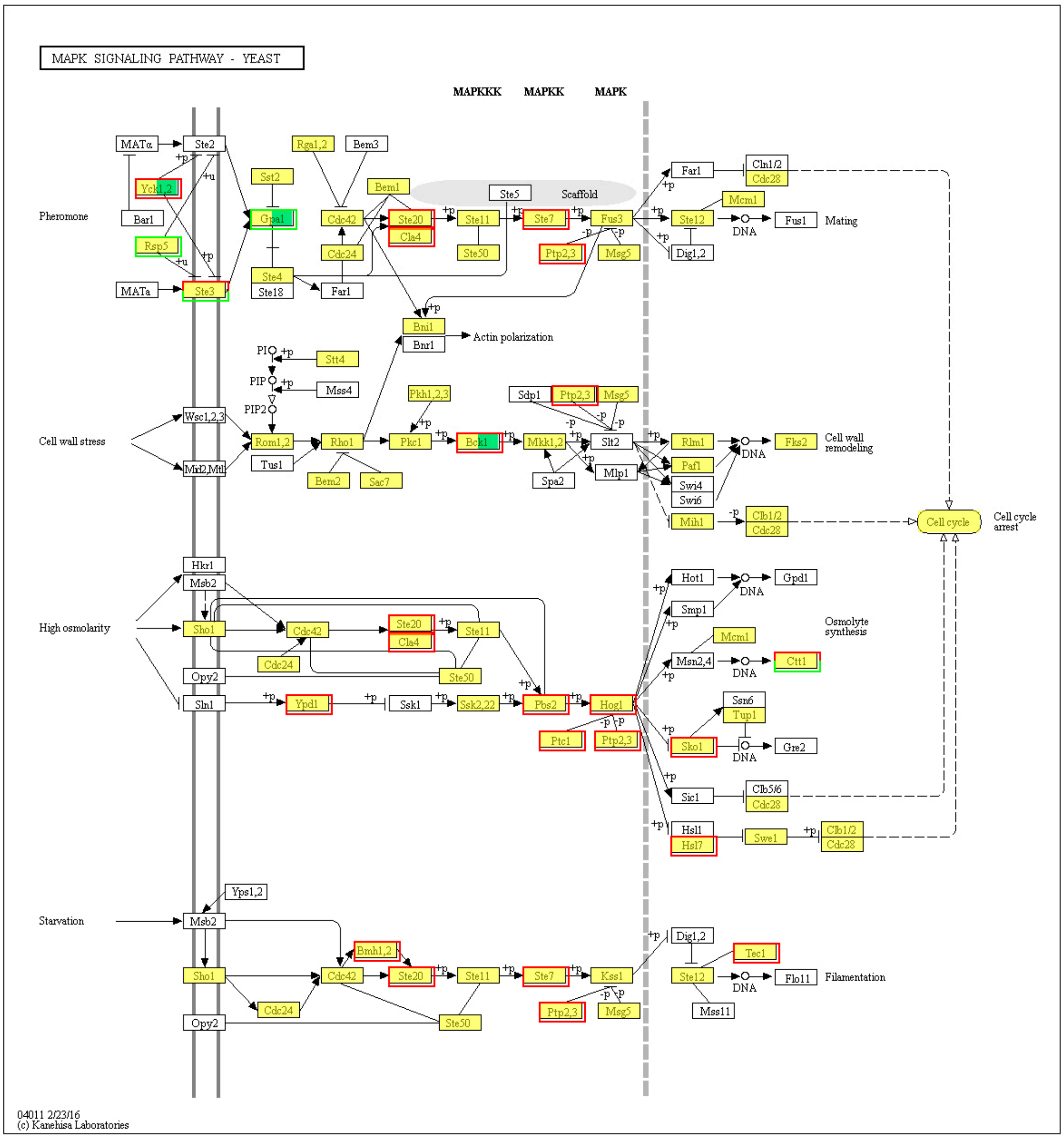

Figure S16. The vegetative-to-reproductive transition stage related pathway-Mitogen-activated protein kinases (MAPK) signaling pathway (derived from KEGG map04011) [1-3] was identified by KEGG annotation. The red and green boxes indicate that the up-regulation and down-regulation genes.

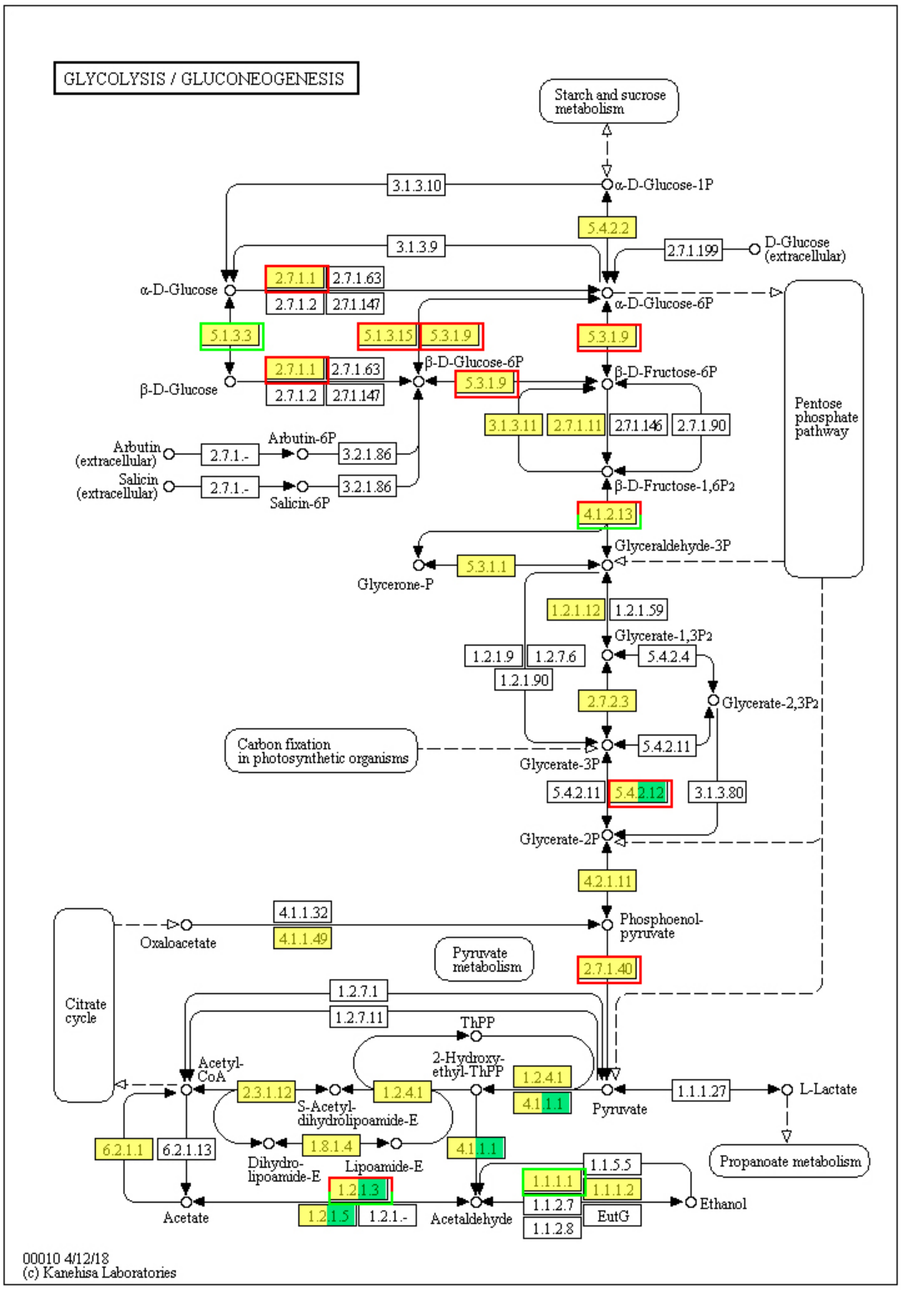

Figure S17. The reproductive growth stage related pathway-Glycolysis (derived from KEGG map00010) [1-3] was identified by KEGG annotation. The red and green boxes indicate that the up-regulation and down-regulation genes.

Supplementary Tables

Table S1. Primer sets used for quantitative real-time PCR.

Primer name	*Sequence (5'-3')*
tubulin-F	*TTCTGTTCGATCGGGTCCAC*
tubulin-R	*CTTTCGCCCAGTTGTTTCCC*
GAPDH-F	*GCACCCATGTTTGTATGCGG*
GAPDH-R	*GGAGCCAAGCAGTTAGTCGT*
A2498-F	*GGTTTCGCTGTCGCTTTGTT*
A2498-R	*TGAACCGTCGGATGAAGCAA*
A1881-F	*CCCACTTCGACTGCGAGAAA*
A1881-R	*GGCATCCCTTTCATGACGGA*
A6933-F	*GTCGGAAACACATGGTGCAG*
A6933-R	*ACACCCTCCATTGTTAGGCG*
A4619-F	*TCGAACGACTGAAGAAGCGA*
A4619-R	*AATCATCCGTGAGGCCAAGG*
A3758-F	*TCAACCGATTACGGAACGCA*
A3758-R	*CCTGTGAGGCTTCATTGGGT*
A1717-F	*AGATGTGGACAGCGAAAGCA*
A1717-R	*AACGGGTTCAACAGATCGCT*
A0733-F	*GGCTGCACATCCTGTCACT*
A0733-R	*GCAGCGAGTCATTGGGTGAT*
A5568-F	*ATGTCTTGGGAACGTGGTGG*
A5568-R	*TGAATCGCTGACACTACCGC*
A5121-F	*TGTCGGGCTATGGACAACAC*
A5121-R	*GCATGCGAAGCGTGATGATT*
A0367-F	*CGGCGAAGGACCGAATAAGA*
A0367-R	*TTCCACACGGGCTTTGAGTT*
A3021-F	*GGCGATAGTGGTGATGCACT*
A3021-R	*TGATTCGCCGCAGATGAAGT*
A2106-F	*GGGACGTAAAGCCGGACAAT*
A2106-R	*GATGATGCGTTGCTGGTGTC*
A2001-F	*GTGGAGCTGGTCCGAAGAAA*
A2001-R	*TCTCTCTTTGTGCGGCATGT*
A3431-F	*CCATCTCACTCCGGCATCAA*
A3431-R	*ATTCCGCGACCCGAGTAAAG*
A1206-F	*TCATCGCACTCCTCACAGTC*
A1206-R	*CCACTTCTTGCTGGGTTCCA*
A1150-F	*ACTTCGCTAGACAAACGCGA*
A1150-R	*CCATGACGCGAGGTAATCGT*
A1482-F	*TGGGCTATGGTTCACACGAC*
A1482-R	*AGTGATGCCACCTGGTTGAG*
A0126-F	*AAGCAGCATCGCTCAGAACT*
A0126-R	*TTCCAAGCGTTCCAAATGCG*
A6478-F	*GTCAAAGACGCTGGGAGACA*
A6478-R	*TACGGGAGAGTCGGGTGAAT*

Table S2. Statistical result of interspersed nuclear elements.

Type	*Number*	*Total Length (bp)*	*In Genome (%)*	*Average length (bp)*
LTR	*1,701*	*545,223*	*2.0191*	*324*
DNA	*395*	*26,363*	*0.0976*	*70*
LINE	*444*	*34,240*	*0.1268*	*79*
SINE	*10*	*679*	*0.0025*	*68*
RC	*44*	*7,320*	*0.0271*	*167*
Unknown	*11*	*1,378*	*0.0051*	*125*
Total	*2,605*	*611,966*	*2.2663*	*240*

LTR: long terminal repeat; DNA: DNA transposon; LINEs : Long inter spersed nuclear elements; SINEs: Short interspersed nuclear elements; RC: rolling circle.

Table S3. Statistical result of tandem repeats.

Type	*Number*	*Repeat Size (bp)*	*Total Length (bp)*	*In Genome (%)*
Tandem repeat	*2,362*	*1~509*	*133,595*	*0.4947*
Minisatellite DNA	*2,027*	*10~60*	*93,368*	*0.3458*
Microsatellite DNA	*132*	*2~6*	*6,181*	*0.0229*

Table S4. Statistical result of non-coding RNA.

Type	*Number*	*Average length (bp)*	*Total length (bp)*
tRNA	*93*	*85*	*7,945*
5s(denovo)	*1*	*114*	*114*
5.8s(denovo)	*0*	*0*	*0*
18s(denovo)	*4*	*1,841*	*7,365*
28s(denovo)	*2*	*4,386*	*8,772*
sRNA	*0*	*0*	*0*
snRNA	*11*	*117*	*1,297*
miRNA	*0*	*0*	*0*

Table S5. Genomic features of *F. luteovirens* and other 5 selected medicinal fungi

Species	*Floccularia luteovirens*	*Hericium erinaceus*	*Ganoderma lucidum*	*Antrodia cinnamomea*	*Tremella mesenterica*	*Inonotus baumii*
Genome Coverage	190x	200x	440x	878x	7.4x	186x
No. of Scaffolds	23	519	82	360	45	217
Genome Size (Mb)	27	39.35	43.3	32.15	28.64	31.64
GC Content of genome (%)	43.54	53.14	55.9	50.6	46.7	47.6
Gene Number	7068	9895	16113	9254	8313	8455

Table S6. Functional Annotation results.

Functional Annotation	*Counts*
Genes assigned to GO	*4680*
Genes assigned to KEGG	*5854*
Genes assigned to KOG	*1532*
Genes assigned to NR	*6136*
Genes assigned to TCDB	*286*
Genes assigned to Pfam	*4680*
Genes assigned to Swiss-Prot	*1973*
Genes assigned to CAZy	*329*

Table S7. Classification of putative carbohydrate metabolism proteins of *Floccularia luteovirens* and other edible fungi or model fungi based on CaZy database [4-6].

Species	*Ectomycorrhizal fungi*		*White-rot fungi*				*Brown-rot fungi*	
	Floccularia luteovirens	*Laccaria bicolor*	*Lentinula edodes*	*Flammulina velutipes*	*Ganoderma lucidum*	*Phanerochaete chrysosporium*	*Antrodia cinnamomea*	*Postia placenta*
GH	*159*	*168*	*245*	*193*	*288*	*190*	*78*	*144*
GT	*58*	*87*	*75*	*85*	*70*	*65*	*54*	*75*
PL	*8*	*7*	*9*	*23*	*10*	*4*	*1*	*6*
CE	*18*	*17*	*31*	*91*	*30*	*16*	*19*	*10*
CBM	*54*	*24*	*58*	*40*	*53*	*47*	*59*	
AA1	*14*	*8*	*14*	*3*	*13*	*0*	*15*	*3*
AA2	*6*	*1*	*9*	*3*	*8*	*16*		*0*
AA3	*22*	*5*	*26*	*23*	*5*	*8*	*3*	*7*
AA4	*0*	*0*	*2*	*0*	*0*	*0*	*0*	*0*
AA5	*3*	*9*	*5*	*6*	*9*	*7*	*4*	*2*
AA6	*3*	*2*	*2*	*2*	*1*	*4*	*0*	*1*
AA7	*1*	*0*	*15*	*15*	*0*	*0*	*0*	*0*
AA8	*1*	*0*	*1*	*1*	*2*	*2*	*0*	*0*
AA9	*8*	*0*	*12*	*16*	*0*	*0*	*4*	*2*
AA10	*0*	*0*	*0*	*0*	*0*	*0*	*0*	*0*
AA11	*0*		*0*	*0*	*0*	*0*	*0*	*0*

GH: Glycoside Hydrolases, GT: Glycosyl Transferases, PL: Polysaccharide Lyases, CE: Carbohydrate Esterases, CBM: Carbohydrate-Binding Modules, AA: Auxiliary

Activities, AA1: laccases, ferroxidases and laccase-like multicopper oxidases. AA2: class II ligninmodifying peroxidases. AA3: flavin-adenine dinucleotide (FAD)-binding domain. AA4: vanillyl-alcohol oxidase. AA5: copper radical oxidases family. AA6: benzoquinone reductases. AA7: glucooligosaccharide oxidase. AA8: Iron reductase domain. AA9-AA11: cooper-dependent lytic polysaccharide monooxygenases (LPMOs).

Table S8. Statistical result of P450 genes

Gene id	*Identity*	*E-value*	*Subject id*	*P450 Class Name*	*P450 Name*
A1280	*47.8*	*1.90E-79*	*CNAG_06628T0*	*Undeterminded*	*Undeterminded*
A1300	*40.8*	*7.40E-98*	*estExt_Genemark1.C_4_t10229*	*E-classP450,groupI*	*P450_052*
A1306	*49.1*	*8.50E-80*	*CNAG_06628T0*	*Undeterminded*	*Undeterminded*
A1333	*53.9*	*1.80E-89*	*estExt_fgenesh3_pg.C_130101*	*E-classP450,groupIV*	*P450_Pp023*
A1353	*53.5*	*3.00E-76*	*g12727.t1*	*E-classP450,groupI*	*P450_024*
A1364	*60.4*	*1.30E-79*	*g12727.t1*	*E-classP450,groupI*	*P450_024*
A1409	*56.2*	*1.00E-147*	*g12727.t1*	*E-classP450,groupI*	*P450_024*
A1475	*62.2*	*5.70E-179*	*fgenesh1_pm.LG_6_*	*E-classP450,groupIV*	*Undeterminded*
A1506	*44.9*	*1.10E-115*	*g5176.t1*	*E-classP450,groupI*	*P450_091*
A1512	*63.3*	*2.70E-225*	*g20224.t1*	*E-classP450,groupIV*	*P450_008*
A1586	*44.7*	*1.60E-108*	*Lacbi1.Eugenev2.0000380190*	*E-classP450,groupI*	*Undeterminded*
A1761	*62.3*	*6.00E-145*	*Lacbi1.eu2.Lbscf0001g03860*	*E-classP450,groupIV*	*Undeterminded*
A1762	*79*	*2.30E-205*	*Lacbi1.estExt_GeneWisePlus_worm.C_10798*	*E-classP450,groupI*	*Undeterminded*
A1845	*43.7*	*2.10E-112*	*estExt_Genewise1Plus.C_180267*	*E-classP450,groupI*	*P450_Sl7.9012*
A2209	*41.2*	*2.60E-62*	*CNAG_06628T0*	*Undeterminded*	*Undeterminded*
A2247	*50.6*	*4.10E-153*	*g12727.t1*	*E-classP450,groupI*	*P450_024*
A2435	*40*	*6.10E-121*	*AO090701000601*	*CytochromeP450*	*P450_Ao161*
A2506	*40.4*	*1.30E-115*	*AO090701000601*	*CytochromeP450*	*P450_Ao161*
A2549	*41*	*5.30E-12*	*PGUG_03908.1*	*CytochromeP450*	*P450_Cgu005*
A2593	*73.3*	*1.60E-137*	*estExt_fgenesh1_pm.C_12_t10450*	*E-classP450,groupI*	*Undeterminded*
A2613	*45.9*	*5.40E-125*	*AFL2G_07275*	*Undeterminded*	*P450_Afl090*
A2618	*50.3*	*6.60E-121*	*AFL2G_07275*	*Undeterminded*	*P450_Afl090*
A2733	*48.1*	*1.40E-71*	*fgenesh1_kg.11_*	*E-classP450,groupI*	*P450_Ds048*
A2764	*42*	*3.10E-118*	*AFL2G_07275*	*Undeterminded*	*P450_Afl090*
A2814	*79.9*	*3.70E-239*	*CC1G_02050*	*Undeterminded*	*P450_Cc020*
A2856	*54.6*	*3.50E-91*	*CC1G_02050*	*Undeterminded*	*P450_Cc020*
A2867	*67.9*	*9.00E-62*	*g18438.t1*	*E-classP450,groupI*	*P450_001*

A2975	*49.9*	*3.20E-133*	*estExt_Genewise1.C_120390*	*E-classP450,CYP3A*	*P450_Abi074*
A3011	*48.8*	*2.10E-55*	*estExt_Genewise1Plus.C_180253*	*E-classP450,groupI*	*Undeterminded*
A3043	*44.6*	*5.90E-183*	*EEA23218.1*	*E-classP450,groupI*	*P450_Pm070*
A3261	*48.6*	*4.90E-114*	*fgenesh3_pg.83__41*	*CytochromeP450*	*P450_Pp164*
A3481	*43.9*	*9.30E-246*	*HCB03452.1*	*E-classP450,CYP2D*	*P450_Hc024*
A3560	*40.2*	*1.40E-98*	*Pa_7_5740*	*E-classP450,groupIV*	*P450_Pa074*
A3706	*50*	*1.60E-104*	*EEA26647.1*	*E-classP450,groupI*	*P450_Pm001*
A3707	*48.7*	*1.60E-104*	*EEA26647.1*	*E-classP450,groupI*	*P450_Pm001*
A3718	*61.4*	*5.80E-190*	*g18262.t1*	*E-classP450,groupI*	*P450_238*
A3743	*55*	*4.00E-161*	*genemark.102_g*	*E-classP450,groupI*	*P450_Po9085*
A3745	*62.3*	*1.20E-195*	*genemark.102_g*	*E-classP450,groupI*	*P450_Po9085*
A3746	*49.3*	*9.70E-28*	*fgenesh1_kg.6_*	*E-classP450,groupI*	*P450_048*
A3795	*55.2*	*0.00E+00*	*g14592.t1*	*E-classP450,groupI*	*P450_057*
A4055	*76.4*	*2.70E-248*	*Lacbi1.fgenesh3_pg.C_scaffold_4000074*	*E-classP450,groupI*	*Undeterminded*
A4057	*68.7*	*0.00E+00*	*estExt_fgenesh2_pg.C_10473*	*E-classP450,groupI*	*P450_Sc006*
A4069	*56.3*	*8.00E-136*	*CC1G_15555*	*P450,CYP52*	*P450_Cc136*
A4070	*60*	*2.30E-127*	*Lacbi1.eu2.Lbscf0061g00300*	*E-classP450,groupI*	*Undeterminded*
A4071	*55.7*	*1.30E-165*	*CC1G_15555*	*P450,CYP52*	*P450_Cc136*
A4115	*47.7*	*2.60E-128*	*PCON_06884m.01*	*Undeterminded*	*Undeterminded*
A4152	*60*	*1.10E-170*	*estExt_fgenesh3_kg.C_LG_9_t20137*	*E-classP450,groupI*	*Undeterminded*
A4428	*45*	*3.20E-97*	*Hetan1.EuGene10000586*	*E-classP450,groupI*	*P450_Ha127*
A4571	*43.1*	*2.40E-119*	*Fompi1.gm1.5568_g*	*E-classP450,groupI*	*P450_Fp130*
A4576	*52*	*3.30E-96*	*e_gw1.5.1517.1*	*E-classP450,groupI*	*P450_Cp205*
A4582	*66.5*	*3.10E-200*	*eu2.Lbscf0025g02180*	*P450,CYP52*	*P450_Lb033*
A4586	*46.4*	*2.10E-131*	*fgenesh1_kg.1_*	*E-classP450,groupI*	*P450_Ds006*
A4596	*71.6*	*3.60E-182*	*Lacbi1.Eugenev2.0000250149*	*E-classP450,groupI*	*Undeterminded*
A4602	*67.3*	*1.90E-202*	*eu2.Lbscf0025g02180*	*P450,CYP52*	*P450_Lb033*
A4603	*52.1*	*2.20E-131*	*genemark.2466_g*	*P450,CYP52*	*P450_Po9061*
A4604	*52.5*	*6.60E-165*	*g12727.t1*	*E-classP450,groupI*	*P450_024*
A4657	*53.4*	*2.70E-168*	*g12727.t1*	*E-classP450,groupI*	*P450_024*
A4793	*66.9*	*2.80E-216*	*fgenesh1_kg.5_*	*E-classP450,groupI*	*P450_011*

				V	
A4894	*47.8*	*2.00E-93*	*Lacbi1.eu2.Lbscf0008g02830*	*CytochromeP450*	*Undeterminded*
A5007	*61.3*	*4.30E-131*	*Hetan1.estExt_Genewise1Plus.C_80519*	*P450,CYP52*	*P450_Ha109*
A5009	*62.5*	*2.70E-250*	*g20224.t1*	*E-classP450,groupIV*	*P450_008*
A5074	*66.1*	*5.60E-203*	*fgenesh3_kg.LG_1_/3_Confidence_0.750*	*E-classP450,groupI*	*Undeterminded*
A5093	*61.5*	*2.30E-79*	*g13734.t1*	*CytochromeP450*	*P450_140*
A5193	*52*	*1.70E-146*	*estExt_fgenesh1_pg.C_00003_t20110*	*E-classP450,groupI*	*P450_Gt099*
A5253	*42.7*	*4.40E-123*	*AFL2G_00089*	*E-classP450,groupI*	*P450_Afl003*
A5298	*55.8*	*1.40E-229*	*g17951.t1*	*CytochromeP450*	*P450_232*
A5347	*75.3*	*6.40E-183*	*fgenesh1_pg.8_*	*CytochromeP450*	*Undeterminded*
A5361	*52.5*	*1.70E-206*	*g16550.t1*	*E-classP450,groupI*	*P450_217*
A5523	*48.8*	*1.20E-264*	*maker-scaffold_6-exonerate_est2genome-gene-2.14-mRNA-1*	*CytochromeP450*	*P450_056*
A5636	*42.7*	*9.70E-63*	*EEB89366.1*	*E-classP450,groupI*	*P450_MP270*
A5640	*42.3*	*6.30E-56*	*gm1.10056_g*	*E-classP450,groupIV*	*P450_Gt097*
A5673	*47.1*	*1.50E-142*	*gm1.10052_g*	*E-classP450,groupI*	*P450_Gt096*
A5676	*42.3*	*8.00E-117*	*Hetan1.fgenesh2_pm.C_scaffold_3000526*	*E-classP450,groupI*	*P450_Ha048*
A5684	*70.2*	*1.10E-208*	*Lacbi1.Eugenev2.0000020149*	*E-classP450,groupI*	*Undeterminded*
A6013	*51.4*	*1.10E-159*	*fgenesh1_pm.7_*	*Undeterminded*	*P450_Wc136*
A6027	*61.5*	*1.60E-72*	*fgenesh1_pm.4_*	*E-classP450,groupI*	*P450_Ds014*
A6110	*74.4*	*5.50E-241*	*estExt_Genewise1.C_LG_3_t90260*	*E-classP450,groupIV*	*Undeterminded*
A6130	*50*	*1.70E-106*	*estExt_fgenesh1_pg.C_00003_t20110*	*E-classP450,groupI*	*P450_Gt099*
A6131	*47.2*	*2.50E-140*	*estExt_fgenesh1_pg.C_00003_t20110*	*E-classP450,groupI*	*P450_Gt099*
A6181	*59.8*	*6.00E-181*	*eu2.Lbscf0061g00290*	*Pisatindemethylase-like*	*P450_Lb029*
A6303	*61*	*5.30E-226*	*EfO2.075130.1*	*Undeterminded*	*P450_EF032*
A6336	*50.8*	*3.30E-173*	*Lacbi1.eu2.Lbscf0025g01890*	*E-classP450,groupI*	*Undeterminded*

A6439	65.7	4.80E-225	Hanno_08615	E-classP450,groupI	P450_Ha0153
A6441	66	2.10E-211	fgenesh2_pm.12_	E-classP450,CYPI A	P450_Abi083
A6491	68.5	5.50E-181	CC1G_06525	E-classP450,groupI	P450_Cc060
A6525	56.1	0.00E+00	Lacbi1.eu2.Lbscf0068g0015 0	CytochromeP450	Undeterminded
A6641	55.7	4.80E-184	estExt_Genewise1.C_LG_6_ t40474	E-classP450,groupI	Undeterminded
A6695	52.3	7.10E-29	Hanno_01438	E-classP450,groupI	P450_Ha0178
A6698	47.3	3.50E-77	fgenesh1_kg.9_	E-classP450,groupI	P450_Gs162
A6699	43.9	1.60E-122	fgenesh1_kg.1_	E-classP450,groupI	P450_Ds006
A6700	45.8	1.50E-108	fgenesh1_kg.6_	E-classP450,groupI	P450_048
A6701	44.2	1.90E-112	e_gw1.5.300.1	E-classP450,groupI	P450_Po9036
A6702	43.2	2.00E-106	fgenesh1_kg.6_	E-classP450,groupI	P450_048
A6703	44.3	5.60E-118	fgenesh1_kg.9_	E-classP450,groupI	P450_Gs162
A6704	46.6	2.60E-122	fgenesh1_kg.9_	E-classP450,groupI	P450_Gs162
A6705	46.2	1.20E-102	fgenesh1_kg.9_	E-classP450,groupI	P450_Gs162
A6709	46.6	2.60E-66	CH063_09276T0	E-classP450,groupI	P450_147
A6775	57.4	2.90E-164	Lacbi1.Eugenev2.000009038 8	E-classP450,groupI	Undeterminded
A6793	56.1	1.30E-162	CC1G_11027	P450,CYP52	P450_Cc103
A6817	54.4	2.60E-116	fgenesh1_pg.C_scaffold_900 0455	E-classP450,groupI	P450_Pc036
A6850	50.3	3.70E-82	CNAG_06628T0	Undeterminded	Undeterminded
A6853	57.7	1.00E-166	CNAG_06628T0	Undeterminded	Undeterminded
A6914	55.9	7.70E-199	CC1G_02244	P450,CYP52	P450_Cc022
A6941	45.8	1.20E-114	AFL2G_07275	Undeterminded	P450_Afl090
A7000	45.3	6.80E-122	AFL2G_07275	Undeterminded	P450_Afl090
A7010	58.5	0.00E+00	Lacbi1.eu2.Lbscf0002g0332 0	CytochromeP450	Undeterminded
A7030	45.5	5.20E-102	fgenesh1_pg.8_	CytochromeP450	Undeterminded
A7058	53.9	2.50E-112	CC1G_06525	E-classP450,groupI	P450_Cc060
A0023	50	4.40E-53	fgenesh1_kg.4_	E-classP450,groupI	P450_077
A0025	50.9	5.90E-113	estExt_fgenesh1_pg.C_3012 5	E-classP450,groupI	P450_Sl7.9087
A0026	49.7	1.50E-125	estExt_fgenesh1_pg.C_3012 5	E-classP450,groupI	P450_Sl7.9087
A0027	51.7	2.80E-127	estExt_fgenesh1_pg.C_3012 5	E-classP450,groupI	P450_Sl7.9087
A0028	43.6	8.10E-35	fgenesh1_kg.C_scaffold_100	E-classP450,groupI	P450_Sc008

			0183		
A0053	*83.8*	*1.90E-248*	*fgenesh1_pm.29_*	*E-classP450,groupI*	*P450_Po9137*
A0108	*50.6*	*6.30E-105*	*g19105.t1*	*E-classP450,groupI*	*P450_107*
A0113	*76.4*	*5.20E-241*	*fgenesh1_pm.29_*	*E-classP450,groupI*	*P450_Po9137*
A0124	*61.6*	*2.20E-135*	*e_gw1.10.2124.1*	*E-classP450,groupI*	*Undeterminded*
A0164	*61.9*	*4.70E-170*	*estExt_fgenesh1_kg.C_0001 30084*	*E-classP450,groupI*	*P450_Gt104*
A0169	*53.2*	*1.60E-91*	*g12555.t1*	*E-classP450,groupI*	*P450_022*
A0174	*48.4*	*2.10E-128*	*fgenesh1_kg.6_*	*E-classP450,groupI*	*P450_048*
A0185	*42.2*	*9.40E-116*	*fgenesh1_kg.9_*	*E-classP450,groupI*	*P450_Gs162*
A0218	*49.5*	*1.40E-146*	*estExt_fgenesh3_kg.C_LG_2 _t40107*	*E-classP450,groupI*	*Undeterminded*
A0220	*51.2*	*2.70E-80*	*Lacbi1.Eugenev2.000008047 2*	*E-classP450,groupI*	*Undeterminded*
A0234	*77.8*	*3.40E-212*	*Lacbi1.Eugenev2.000017026 3*	*E-classP450,groupI*	*Undeterminded*
A0236	*58.2*	*2.50E-188*	*Lacbi1.Eugenev2.000017026 3*	*E-classP450,groupI*	*Undeterminded*
A0272	*44*	*1.00E-86*	*fgenesh1_kg.9_*	*E-classP450,groupI*	*P450_Gs162*
A0274	*45.2*	*5.60E-109*	*fgenesh1_kg.9_*	*E-classP450,groupI*	*P450_Gs162*
A0275	*46.5*	*1.50E-82*	*e_gw1.5.300.1*	*E-classP450,groupI*	*P450_Po9036*
A0277	*44.8*	*5.40E-70*	*fgenesh1_kg.3_*	*E-classP450,groupI*	*P450_Ds011*
A0348	*47.3*	*4.40E-119*	*estExt_Genewise1.C_LG_6_ t40474*	*E-classP450,groupI*	*Undeterminded*
A0350	*46.1*	*5.30E-114*	*estExt_Genewise1.C_LG_6_ t40474*	*E-classP450,groupI*	*Undeterminded*
A0363	*54.4*	*1.50E-86*	*estExt_fgenesh1_kg.C_0001 30084*	*E-classP450,groupI*	*P450_Gt104*
A0365	*56.8*	*1.90E-135*	*estExt_fgenesh3_kg.C_LG_9 _t20137*	*E-classP450,groupI*	*Undeterminded*
A0366	*45*	*5.00E-101*	*CC1G_08039*	*E-classP450,groupI*	*P450_Cc072*
A0374	*43.4*	*1.80E-22*	*Hanno_01438*	*E-classP450,groupI*	*P450_Ha0178*
A0380	*57.5*	*6.00E-173*	*estExt_fgenesh1_kg.C_0001 30084*	*E-classP450,groupI*	*P450_Gt104*
A0381	*57.8*	*2.70E-166*	*estExt_fgenesh1_kg.C_0001 30084*	*E-classP450,groupI*	*P450_Gt104*
A0411	*47.9*	*5.40E-25*	*estExt_fgenesh3_pg.C_3139 0001*	*P450,CYP52*	*P450_Pp342*
A0429	*40.9*	*5.20E-53*	*EEB89366.1*	*E-classP450,groupI*	*P450_MP270*
A0442	*47.8*	*1.70E-127*	*estExt_Genewise1.C_1_t700*	*CytochromeP450*	*P450_Cp151*

			74		
A0508	*40.2*	*1.40E-104*	*fgenesh1_kg.11_*	*E-classP450,groupI*	*P450_Ds048*
A0525	*49.4*	*1.40E-117*	*Lacbi1.Eugenev2.000033009 6*	*E-classP450,groupI V*	*Undeterminded*
A0539	*70*	*1.40E-211*	*Lacbi1.eu2.Lbscf0033g0077 0*	*E-classP450,groupI*	*Undeterminded*
A0615	*78.3*	*2.80E-254*	*Lacbi1.estExt_fgenesh2_pm. C_120018*	*E-classP450,groupI*	*Undeterminded*
A1037	*43.4*	*2.00E-112*	*estExt_Genewise1Plus.C_05 1678*	*E-classP450,groupI*	*P450_Ha035*

Table S9. Transporters in *F. luteovirens*.

Transporter family	*Family discription*	*Gene number*
1.A.1	*The Voltage-gated Ion Channel (VIC) Superfamily*	*1*
1.A.101	*The Peroxisomal Pore-forming Pex11 (Pex11) Family*	*1*
1.A.11	*The Ammonium Transporter Channel (Amt) Family*	*3*
1.A.17	*The Calcium-Dependent Chloride Channel (Ca-ClC) Family*	*1*
1.A.33	*The Cation Channel-forming Heat Shock Protein-70 (Hsp70) Family*	*4*
1.A.77	*The Mg^{2+} /Ca^{2+} Uniporter (MCU) Family*	*1*
1.A.8	*The Major Intrinsic Protein (MIP) Family*	*4*
1.A.88	*The Fungal Potassium Channel (F-Kch) Family*	*1*
1.B.69	*The Peroxysomal Membrane Porin 4 (PxMP4) Family*	*1*
1.F.1	*The Synaptosomal Vesicle Fusion Pore (SVF-Pore) Family*	*1*
1.I.1	*The Eukaryotic Nuclear Pore Complex (E-NPC) Family*	*19*
2.A.1	*The Major Facilitator Superfamily (MFS)*	*16*
2.A.105	*The Mitochondrial Pyruvate Carrier (MPC) Family*	*2*
2.A.106	*The Ca^{2+} :H^{+} Antiporter-2 (CaCA2) Family*	*1*
2.A.108	*The Iron/Lead Transporter (ILT) Family*	*2*
2.A.126	*The Fatty Acid Exporter (FAX) Family*	*1*
2.A.17	*The Proton-dependent Oligopeptide Transporter (POT/PTR) Family*	*3*
2.A.19	*The Ca 2+ :Cation Antiporter (CaCA) Family*	*3*
2.A.29	*The Mitochondrial Carrier (MC) Family*	*15*
2.A.3	*The Amino Acid-Polyamine-Organocation (APC) Family*	*6*
2.A.31	*The Anion Exchanger (AE) Family*	*1*
2.A.36	*The Monovalent Cation:Proton Antiporter-1 (CPA1) Family*	*4*
2.A.37	*The Monovalent Cation:Proton Antiporter-2 (CPA2) Family*	*1*
2.A.38	*The K^{+} Transporter (Trk) Family*	*1*
2.A.4	*The Cation Diffusion Facilitator (CDF) Family*	*4*
2.A.40	*The Nucleobase/Ascorbate Transporter (NAT) or Nucleobase:Cation Symporter-2 (NCS2) Family*	*1*
2.A.49	*The Chloride Carrier/Channel (ClC) Family*	*1*
2.A.5	*The Zinc (Zn^{2+})-Iron (Fe^{2+}) Permease (ZIP) Family*	*2*
2.A.50	*The Glycerol Uptake (GUP) Family*	*1*
2.A.52	*The Ni^{2+} -Co^{2+} Transporter (NiCoT) Family*	*1*
2.A.53	*The Sulfate Permease (SulP) Family*	*1*
2.A.55	*The Metal Ion (Mn 2+ -iron) Transporter (Nramp) Family*	*2*
2.A.59	*The Arsenical Resistance-3 (ACR3) Family*	*1*
2.A.6	*The Resistance-Nodulation-Cell Division (RND) Superfamily*	*1*
2.A.66	*The Multidrug/Oligosaccharidyl-lipid/Polysaccharide (MOP) Flippase*	*1*

	Superfamily	
2.A.67	*The Oligopeptide Transporter (OPT) Family*	*4*
2.A.7	*The Drug/Metabolite Transporter (DMT) Superfamily*	*4*
2.A.89	*The Vacuolar Iron Transporter (VIT) Family*	*1*
2.A.96	*The Acetate Uptake Transporter (AceTr) Family*	*2*
2.A.97	*The Mitochondrial Inner Membrane K^+/H^+ and Ca^{2+}/H^+ Exchanger (LetM1) Family*	*1*
2.D.1	*The PI4P/PS Counter Transporter (P/P-CT) Family*	*1*
3.A.1	*The ATP-binding Cassette (ABC) Superfamily*	*9*
3.A.16	*The Endoplasmic Reticular Retrotranslocon (ER-RT) Family*	*7*
3.A.18	*The Nuclear mRNA Exporter (mRNA-E) Family*	*4*
3.A.19	*The TMS Recognition/Insertion Complex (TRC) Family*	*1*
3.A.2	*The H^+- or Na^+-translocating F-type, V-type and A-type ATPase (F-ATPase) Superfamily*	*14*
3.A.20	*The Peroxisomal Protein Importer (PPI) Family*	*5*
3.A.23	*The Type VI Symbiosis/Virulence Secretory Pathway (VISP) Family*	*2*
3.A.25	*The Symbiont-specific ERAD-like Machinery (SELMA) Family*	*1*
3.A.26	*The Plasmodium Translocon of Exported proteins (PTEX) Family*	*1*
3.A.27	*The C-terminal Tail-Anchored Membrane Protein Biogenesis/ Insertion Complex-2 (TAMP-B2) Family*	*1*
3.A.28	*The AAA-ATPase, Bcs1 (Bcs1) Family*	*5*
3.A.29	*The Mitochondrial Inner Membrane i-AAA Protease Complex (MIMP) Familly*	*2*
3.A.3	*The P-type ATPase (P-ATPase) Superfamily*	*13*
3.A.5	*The General Secretory Pathway (Sec) Family*	*5*
3.A.8	*The Mitochondrial Protein Translocase (MPT) Family*	*3*
3.D.1	*The H^+ or Na^+-translocating NADH Dehydrogenase (NDH) Family*	*16*
3.D.10	*The Prokaryotic Succinate Dehydrogenase (SDH) Family*	*1*
3.D.2	*The Proton-translocating Transhydrogenase (PTH) Family*	*1*
3.D.3	*The Proton-translocating Quinol:Cytochrome c Reductase (QCR) Superfamily*	*2*
3.D.4	*The Proton-translocating Cytochrome Oxidase (COX) Superfamily*	*5*
4.D.1	*The Putative Vectorial Glycosyl Polymerization (VGP) Family*	*2*
4.D.3	*The Glycan Glucosyl Transferase (OpgH) Family*	*2*
5.B.1	*The Phagocyte (gp91 phox) NADPH Oxidase Family*	*1*
8.A.104	*The 5'-AMP-activated protein kinase (AMPK) Family*	*1*
8.A.11	*The Immunophilin-like Prolyl:peptidyl Isomerase Regulator (I-PPI) Family*	*2*
8.A.21	*The Stomatin/Podocin/Band 7/Nephrosis.2/SPFH (Stomatin) Family*	*1*
8.A.27	*The CDC50 P-type ATPase Lipid Flippase Subunit (CDC50) Family*	*1*
8.A.30	*The Nedd4-Family Interacting Protein-2 (Nedd4) Family*	*2*

8.A.32	*The β-Amyloid Cleaving Enzyme (BACE1) Family*	*1*
8.A.34	*The Endophilin (Endophilin) Family*	*2*
8.A.5	*The Voltage-gated K + Channel β-subunit (Kvβ) Family*	*3*
8.A.61	*The Endoplasmic Reticulum-derived Vesicle Protein, Erv14 (Erv14) Family*	*1*
8.A.63	*The Sigma Non-opioid Intracellular Recpetor, (S1R) Family*	*1*
8.A.67	*The Os-9 Quality Control (ERAD) Protein (Os-9) Family*	*1*
8.A.82	*The Calmodulin Calcium Binding Protein (Calmodulin) Family*	*4*
8.A.92	*The G-Protein αβγ Complex (GPC) Family*	*2*
8.A.98	*The 14-3-3 protein (14-3-3) Family*	*2*
9.A.15	*The Autophagy-related Phagophore-formation Transporter (APT) Family*	*4*
9.A.48	*The Unconventional Protein Secretion (UPS) System*	*1*
9.A.50	*The Nuclear t-RNA exporter (t-Exporter) Family*	*1*
9.A.6	*The ATP Exporter (ATP-E) Family*	*1*
9.A.60	*The Small Nuclear RNA Exporter (snRNA-E)*	*1*
9.A.63	*The Retromer-dependent Vacuolar Protein Sorting (R-VPS) Family*	*4*
9.A.64	*The SRP-independent Targeting (SND) Family*	*1*
9.A.70	*The Aspartate Amino Transferase (AAT) Family*	*1*
9.B.1	*The Integral Membrane CAAX Protease (CAAX Protease) Family*	*1*
9.B.105	*The Lead Resistance Fusion Protein (PbrBC) Family*	*1*
9.B.106	*The Pock Size-determining Protein (PSDP) Family*	*1*
9.B.119	*The Glycan Synthase, Fks1 (Fks1) Family*	*2*
9.B.12	*The Sensitivity to Sodium or Salt Stress-induced Hydrophobic Peptide (Sna) Family*	*1*
9.B.142	*The Integral membrane Glycosyltransferase family 39 (GT39) Family*	*1*
9.B.143	*The 6 TMS DUF1275/Pf06912 (DUF1275) Family*	*1*
9.B.146	*The Putative Undecaprenyl-phosphate N-Acetylglucosaminyl Transferase (MurG) Family*	*1*
9.B.191	*The Endoplasmic Reticulum Retention Receptor (KDELR) Family*	*1*
9.B.199	*The 4 TMS PF05225 (PF0225) Family*	*1*
9.B.214	*The ER to Golgi Transport Factor (ER/G-TF) Family*	*1*
9.B.27	*The DedA or YdjX-Z (DedA) Family*	*1*
9.B.278	*The Organellar-targeting Adaptor Protein Complex (O-APC) Family*	*6*
9.B.35	*The Putative Thyronine-Transporting Transthyretin (Transthyretin) Family*	*1*
9.B.45	*The Fungal Mating-type Pheromone Receptor (MAT-PR) Family*	*1*
9.B.57	*The Conidiation and Conidial Germination Protein (CCGP) Family*	*1*
9.B.82	*The Endoplasmic Reticulum Retrieval Protein1 (Putative Heavy Metal Transporter) (Rer1) Family*	*1*

Table S10. Putative gene clusters coding for secondary metabolites in *F. luteovirens* C10.

Region	*BGC Type*	*Location*	*From (bp)*	*To (bp)*	*Most similar known cluster/Similarity*
Region 2.1	*NRPS-like*	*Contig2*	*54457*	*94716*	-
Region 2.2	*NRPS-like*	*Contig2*	*378784*	*430821*	-
Region 3.1	*Terpene*	*Contig3*	*407486*	*428367*	-
Region 4.1	*T1PKS*	*Contig4*	*1030652*	*1077591*	*Guadinomine biosynthesis/14%*
Region 6.1	*Terpene*	*Contig6*	*66757*	*90197*	-
Region 6.2	*Terpene*	*Contig6*	*1142364*	*1160270*	-
Region 6.3	*Terpene*	*Contig6*	*1475349*	*1497214*	-
Region 6.4	*T1PKS*	*Contig6*	*1954420*	*1999356*	*Melleolides biosynthesis/100%*
Region 7.1	*Terpene*	*Contig7*	*805334*	*826604*	-
Region 7.2	*Siderophore*	*Contig7*	*1042288*	*1054495*	-
Region 8.1	*NRPS-like*	*Contig8*	*738525*	*782837*	-
Region 9.1	*Terpene*	*Contig9*	*631407*	*652884*	-
Region 10.1	*NRPS-like*	*Contig10*	*948267*	*992814*	-
Region 11.1	*Terpene*	*Contig11*	*305806*	*322161*	-
Region 11.2	*Terpene*	*Contig11*	*954148*	*975488*	-
Region 12.1	*NRPS-like*	*Contig12*	*211839*	*255389*	-

Secondary metabolite types detected by antiSMASH: T1pks (Type I PKS cluster); NRPS (Nonribosomal peptide synthetase cluster). The "similarity" means the percentage of the homologous genes in the query gene cluster that are present in the hit gene cluster. According to the defnition by the antiSMASH, the homologous genes were selected by BLAST e-value<1E-05, 30% minimal sequence identity, shortest BLAST alignment covers over 25%.

Table S11. Taxonomic status of *F. luteovirens*.

Taxonomy	*Classification (Present)*	*Classification (Recommended in this study)*
Domain	*Eukaryota*	*Eukaryota*
Kingdom	*Fungi*	*Fungi*
Phylum	*Basidiomycota*	*Basidiomycota*
Class	*Agaricomycetes*	*Agaricomycetes*
Order	*Agaricales*	*Agaricales*
Family	*Physalacriaceae*	*Tricholomataceae*
Genus	*Armillaria*	*Floccularia*
Species	*Armillaria luteovirens*	*Floccularia luteovirens*

Table S12. Statistics of transcriptome sequencing data.

Sample	*Clean reads*	*Clean bases*	*Error rate(%)*	*Q20(%)*	*Q30(%)*
MF1	*52017332*	*7803901974*	*0.023*	*98.92*	*96.18*
MF2	*50464378*	*7572057083*	*0.0231*	*98.87*	*96.03*
MF3	*48345094*	*7251159310*	*0.0232*	*98.84*	*95.94*
MY1	*46192958*	*6897624102*	*0.024*	*98.45*	*95.08*
MY2	*41706730*	*6237028202*	*0.024*	*98.48*	*95.15*
MY3	*43019518*	*6428838451*	*0.0244*	*98.3*	*94.71*
PR1	*57134800*	*8569013020*	*0.0233*	*98.79*	*95.78*
PR2	*49047642*	*7355982463*	*0.0233*	*98.77*	*95.76*
PR3	*54120672*	*8113401369*	*0.0229*	*98.94*	*96.24*
PR4	*60835486*	*9120970759*	*0.0231*	*98.87*	*96.02*
YF1	*54085920*	*8112693838*	*0.0237*	*98.66*	*95.41*
YF2	*49938676*	*7484767706*	*0.0231*	*98.86*	*96.02*
YF3	*49083070*	*7362344533*	*0.0232*	*98.84*	*95.95*

Table S13. Genes validated by RT-qPCR.

Gene ID	Log2FC (MY-PR)	Gene Name	Gene Description
A2498	3.211468	mei2	Meiosis protein mei2
A1881	3.050398	dmc1	Meiotic recombination protein dmc1
A6933	1.640372	setd2	Histone-lysine N-methyltransferase
A4619	1.340383	cnb	Calcineurin subunit B
A3758	-2.59821	hdac3	Histone deacetylase phd1
A1717	-2.94127	clpB	Heat shock protein 104
A0733	0.909532	Mcm1	transcription factor of the MADS box family
A5568	-0.53136	Rlm1	transcription factor of the MADS box family
A5121	3.785844	bZIP	Basic region leucine zipper;bZIP transcription factor
A0367	3.542702	bHLH	transcription factor
A3021	3.38544	prz1	Transcriptional regulator prz1
A2106	3.097599	steA	transcription factor
A2001	-1.66475	yabby	transcription factor YABBY family protein
A3431	4.355247	exg1	Glucan 1,3-beta-glucosidase
A1206	13.82433	gpi	Ser-Thr-rich glycosyl-phosphatidyl-inositol-anchored membrane family
A1150	-1.34259	nox2	Nicotinamide adenine dinucleotide phosphate oxidases
A1482	-1.37216	nox1	Nicotinamide adenine dinucleotide phosphate oxidases
A4977	-0.15222	snf5	SWI/SNF chromatin-remodeling complex subunit SNF5
A0667	-0.67351	sfh1	Chromatin structure-remodeling complex subunit sfh1
A5507	0.966176	arp9	SWI/SNF and RSC complexes subunit arp9
A6150	-1.96404	pro1	ustiloxin B cluster transcription factor ustR
A6149	-2.72816	pro1	ustiloxin B cluster transcription factor ustR
A6245	0.806948	DavT	5-aminovalerate aminotransferase
A6624	0.756779	car2	Ornithine aminotransferase
A5902	1.478967	priB	Fungal specific transcription factor
A0126	3.220925	Pten	Phosphatidylinositol 3,4,5-trisphosphate 3-phosphatase
A6478	2.313909	wdr24	GATOR complex protein
A1380	0.661727	kras	Ras family

References

1. Kanehisa, M.; Furumichi, M.; Tanabe, M.; Sato, Y.; Morishima, K. KEGG: new perspectives on genomes, pathways, diseases and drugs. *Nucleic Acids Res* **2017**, *45*, D353-D361.
2. Kanehisa, M.; Goto, S. KEGG: kyoto encyclopedia of genes and genomes. *Nucleic Acids Res* **2000**, *28*, 27-30.
3. Kanehisa, M.; Sato, Y.; Kawashima, M.; Furumichi, M.; Tanabe, M. KEGG as a reference resource for gene and protein annotation. *Nucleic acids research* **2015**, *44*, D457-D462.
4. Park, Y.J.; Baek, J.H.; Lee, S.; Kim, C.; Rhee, H.; Kim, H.; Seo, J.S.; Park, H.R.; Yoon, D.E.; Nam, J.Y.; et al. Whole genome and global gene expression analyses of the model mushroom Flammulina velutipes reveal a high capacity for lignocellulose degradation. *PLoS One* **2014**, *9*, e93560.
5. Lu, M.Y.; Fan, W.L.; Wang, W.F.; Chen, T.; Tang, Y.C.; Chu, F.H.; Chang, T.T.; Wang, S.Y.; Li, M.Y.; Chen, Y.H.; et al. Genomic and transcriptomic analyses of the medicinal fungus Antrodia cinnamomea for its metabolite biosynthesis and sexual development. *Proceedings of the National Academy of Sciences of the United States of America* **2014**, *111*, E4743-4752.
6. Martinez, D.; Challacombe, J.; Morgenstern, I.; Hibbett, D.; Schmoll, M.; Kubicek, C.P.; Ferreira, P.; Ruiz-Duenas, F.J.; Martinez, A.T.; Kersten, P.; et al. Genome, transcriptome, and secretome analysis of wood decay fungus Postia placenta supports unique mechanisms of lignocellulose conversion. *Proc Natl Acad Sci U S A* **2009**, *106*, 1954-1959.
